# Concentration Independent Modulation of Local Micromechanics in a Fibrin Gel

**DOI:** 10.1371/journal.pone.0020201

**Published:** 2011-05-23

**Authors:** Maxwell A. Kotlarchyk, Samir G. Shreim, Martha B. Alvarez-Elizondo, Laura C. Estrada, Rahul Singh, Lorenzo Valdevit, Ekaterina Kniazeva, Enrico Gratton, Andrew J. Putnam, Elliot L. Botvinick

**Affiliations:** 1 Department of Biomedical Engineering, University of California Irvine, Irvine, California, United States of America; 2 Beckman Laser Institute and Medical Clinic, University of California Irvine, Irvine, California, United States of America; 3 Laboratory for Fluorescence Dynamics, University of California Irvine, Irvine, California, United States of America; 4 Department of Biomedical Engineering, University of Michigan, Ann Arbor, Michigan, United States of America; 5 Department of Mechanical and Aerospace Engineering and Department of Chemical Engineering and Materials Science, University of California Irvine, Irvine, California, United States of America; 6 Edwards Lifesciences Center for Advanced Cardiovascular Technology, University of California Irvine, Irvine, California, United States of America; Université de Technologie de Compiègne, France

## Abstract

Methods for tuning extracellular matrix (ECM) mechanics in 3D cell culture that rely on increasing the concentration of either protein or cross-linking molecules fail to control important parameters such as pore size, ligand density, and molecular diffusivity. Alternatively, ECM stiffness can be modulated independently from protein concentration by mechanically loading the ECM. We have developed a novel device for generating stiffness gradients in naturally derived ECMs, where stiffness is tuned by inducing strain, while local mechanical properties are directly determined by laser tweezers based active microrheology (AMR). Hydrogel substrates polymerized within 35 mm diameter Petri dishes are strained non-uniformly by the precise rotation of an embedded cylindrical post, and exhibit a position-dependent stiffness with little to no modulation of local mesh geometry. Here we present the device in the context of fibrin hydrogels. First AMR is used to directly measure local micromechanics in unstrained hydrogels of increasing fibrin concentration. Changes in stiffness are then mapped within our device, where fibrin concentration is held constant. Fluorescence confocal imaging and orbital particle tracking are used to quantify structural changes in fibrin on the micro and nano levels respectively. The micromechanical strain stiffening measured by microrheology is not accompanied by ECM microstructural changes under our applied loads, as measured by confocal microscopy. However, super-resolution orbital tracking reveals nanostructural straightening, lengthening, and reduced movement of fibrin fibers. Furthermore, we show that aortic smooth muscle cells cultured within our device are morphologically sensitive to the induced mechanical gradient. Our results demonstrate a powerful cell culture tool that can be used in the study of mechanical effects on cellular physiology in naturally derived 3D ECM tissues.

## Introduction

Hydrogels polymerized from natural, synthetic, or hybrid molecules are commonly used as ECMs for the study of cell-ECM interactions as well as for medically implantable biomaterials and potential scaffolds for tissue regeneration [1], [2]. The design of a hydrogel that mimics the physiological microenvironment requires consideration of a multitude of factors including micromechanical properties [3], [4], [5], biocompatibility, ligand concentration [6], biotransport kinetics, and pore size [7], [8], [9], [10]. Complex interactions between these factors contribute to the transduction of cellular signals, which in turn determines cell survival, proliferation, and phenotype. Uncovering the exact role of stiffness in regulating cells in 3D has proven to be difficult because tuning stiffness in a physiologically relevant system is non-trivial. While the bulk mechanics of 3D matrices can be made effectively more stiff by increasing ECM protein concentration or altering the molecular weight of monomers [6], [10], there is a resulting decrease in mesh pore size, and increase in cellular confinement, resistance to transport, and local concentration of ligand presented to cells cultured within [11]. Protein-polymer hybrid systems such as PEG-fibrinogen or collagen-agarose [12] allow one to tune stiffness independent from bulk ligand concentration [9], [13]. However, the mesh size of these systems is commonly much smaller than their naturally derived protein hydrogel counterparts, thus increasing both resistance to transport and cellular confinement as compared to naturally derived systems. While phenotypic changes have been demonstrated in such systems [14], their relevance is debatable in the context of understanding basic physiology.

Our studies are performed in fibrin, a commonly used naturally occurring viscoelastic biopolymer. Fibrin is the polymerized form of the blood circulating protein fibrinogen, and is the predominant structural component of blood clots that form in response to injury. Fibrin hydrogels exhibit many interesting mechanical properties, including high extensibility [15] and negative compressibility [16], all while maintaining permeability and bulk structural integrity under proteolytic degradation and cellular contraction, making it an ideal substrate for the wound healing process. The molecular basis for fibrin's remarkable physical behavior [15], [17] has been investigated at the scale of individual fibers [18], [19], [20], networks of fibers [18], and within macro-scale hydrogels [21], [22]. A more complete understanding of the role of fibrin's astounding mechanical properties in disease and thrombosis [23], [24], [25], as well its function as a scaffold which drives tissue morphogenesis, will lead to better design strategies for tissue regeneration and engineering.

An interesting property of naturally derived ECMs, such as fibrin, is their tendency to stiffen with stretch. In fact, most tissues persist in a stretched state. This so called “mechanical homeostasis”, where residual stress is present in the absence of any external load, was first demonstrated when excised blood vessels were shown to spring open as they were sliced axially [26]. The residual stress, or prestress, originates from cell contractile forces, which are mediated by actomyosin interactions within cells. Intracellular prestress has been demonstrated by the viscoelastic retraction of photoablated actin stress fibers followed by a decrease in contractility as assessed by traction force microscopy [27], [28], and by the observation that actin stress fibers within cells cultured on pre-stretched membranes buckle when stretch is released [29]. Importantly, cellular traction forces are sufficient to locally deform the ECM [30], whose stiffness is strain-dependent [31]. Therefore, the stiffness of biological ECMs can be tuned by external mechanical loads alone to study the effects of ECM stiffness on cell physiology in 3D.

Here we present a simple cell culture device for generating stiffness gradients within naturally occurring ECMs. Our device generates a gradient in mechanical strain, which induces variable stiffness by the materials' non-linear mechanical properties. We demonstrate our ability to both measure and tune the stiffness within natural fibrin ECM hydrogels within our system, which is both biocompatible and mechanically instructive in the context of aortic smooth muscle cells.

## Materials and Methods

### Fibrin hydrogels

Bovine fibrinogen (Sigma) solutions (2.5, 5, or 10 mg/ml) were prepared in 1× PBS or plain basal media under sterile conditions. In gels prepared for AMR, 20 *µ*l of a 20 *µ*g/ml solution of 2 *µ*m diameter silica beads and 50 *µ*l fetal bovine serum (FBS) were mixed with the sterile-filtered fibrinogen solution for every 1 ml of gel. 1 ml of this final gel solution was added to 20 *µ*l of polymerization-initiating thrombin (Sigma, 50 U/ml) previously aliquoted into a 35 mm glass bottom Petri dish (No. 1.5 glass, Matek). FBS contains factor XIII, a zymogen that contributes to the cross-linking of the fibrin gel when activated to by thrombin. Acellular solutions were left undisturbed for 30 min at room temperature until gelation was complete. For cell-seeded fibrin, cells were incorporated along with microrheology beads and FBS, and gelation was completed in a standard cell culture incubator. To maintain gel hydration, 2 ml of PBS or media were added to the dish after gelation. Fluorescent fibrin gels were constructed with a 1∶10 ratio of fluorescent Alexa-488 fibrinogen (Invitrogen, Carlsbad, CA) to non-fluorescent fibrinogen.

### Parallel plate rheology

Curing and mechanical characterization of the gel was accomplished *in situ* on an AR G2 rheometer (TA Instruments, New Castle, DE) equipped with a Peltier stage and configured with a 20 mm stainless steel parallel plate attachment. The Peltier stage was cooled to 4°C after which 320 µL of fibrinogen solution at 2.5, 5 or 10 mg/ml was injected into a 1050 µm gap between the plate and stage. The edge of the plate was sealed with silicone oil (Arcos Organics, Morris Plains, NJ) to prevent evaporation and the top plate was lowered to 1000 µm. The temperature was increased from 4°C to 37°C over five minutes and then held at 37°C for 45 minutes. Rheology was performed throughout the clotting at 1% strain and 1 rad/sec to confirm full gelation after 45 minutes as indicated by a plateau in the measure of the shear modulus, G′. After gelation, G′ was measured by a frequency sweep from 1 to 100 rad/sec at 1% strain. Strain sweeps were performed from 0.001 to 10 percent strain at a constant frequency of 1 radian/s. Five gels were measured at each concentration of fibrinogen for each measurement set.

### Active microrheology instrumentation

**Figure 1:**
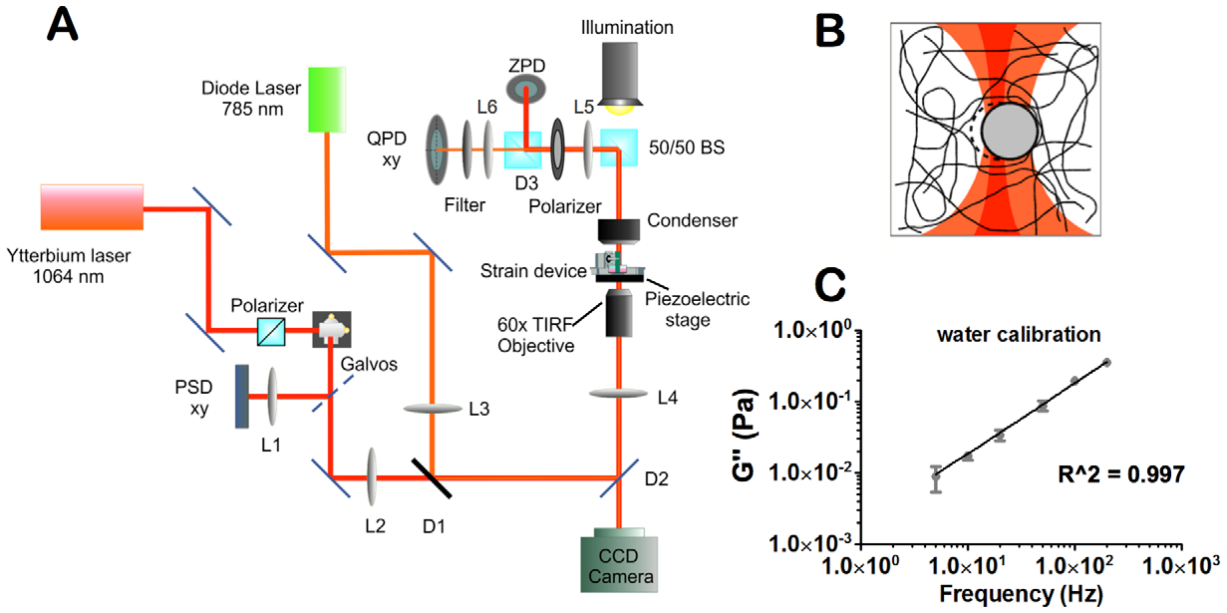
Laser tweezers active microrheology. (A) Active microrheology instrumentation. A trapping laser (1064 nm) and particle position detection laser (785 nm) are combined and focused by the microscope objective into the device, which is mounted in a piezoelectric microscope stage. Forward scattered light is collected by the microscope objective and directed towards the QPD after passing through a filter designed to isolate the 785 nm beam. (B) Illustration of a laser trapped bead bound in a fibrous ECM and oscillated by laser tweezers. Both the trapping (red) and particle detection (orange) laser foci are incident onto the bead. As drawn the trapping beam is applying a leftward force to the bead. (C) Sample AMR loss modulus, G″, measured in water at room temperature reports a viscosity of 1±0.7 cP determined from a linear fit, R^2^ = 0.997.

Our optical instrumentation is an expansion of a passive microrheology instrument previously described [32] and diagramed in Fig. 1A. The instrument is a custom modified Olympus IX81 inverted microscope mounted on a vibration dampening SMART table (Newport). Trapping is achieved by a 1064 nm Ytterbium fiber laser (IPG) steered by XY scanning galvanometer mirrors (Thorlabs). The trapping beam is expanded by lenses L2 (*f* = 400 mm) and L4 (*f* = 500 mm) and focused into the sample by a PlanApo 1.45 NA 60× oil immersion objective (Olympus USA). A 30 mW 785 nm diode laser (World Star Tech) is expanded by lenses L3 (*f* = 150) and L4 (*f* = 500 mm) for particle position detection. The two laser beams are combined by a dichroic beamsplitter D1 (Semrock) and mixed into the microscope imaging path by a short-pass dichroic D2 (Chroma). The trapping beam is partially reflected by a microscope cover glass and focused by L1 (f = 50 mm) onto an XY position sensitive photodiode (PSD, Pacific Silicon Sensor) to monitor the position of the beam during scanning. Forward scattered 785 nm light is refocused by lenses L5 (*f* = 50 mm) and L6 (*f* = 35 mm) onto a quadrant photodiode (QPD, New Focus) positioned conjugate to the back focal plane of our objective lens, while forward scattered 1064 nm light is reflected by a short-pass dichroic D3 (Chroma Technology) to overfill a photodiode (ZPD) for z-position detection (not implemented in this study).

We previously determined 2 um diameter beads were appropriate for microrheology in 2.5, 5, and 10 mg/ml fibrin gels [32]. A sinusoidal optical trap oscillated microbeads embedded within fibrin gels. An oscillating microbead steers the detection laser across the QPD, which outputs three analog signals: diff(*X*), diff(*Y*), and sum. PSD and QPD signals were sampled at 10 kHz for 5 seconds by a multifunction data acquisition board (M-series DAQ, National Instruments). The *X* and *Y* signals were normalized by the sum signal to compensate for small changes in average laser intensity. For each bead, five replicate signals were collected at 5, 10, 20, 50, 100, and 200 Hz for an oscillation amplitude of 60 nm. Hardware and data acquisition were controlled via custom LabVIEW software.

### Active microrheology calibration

PSD signals were calibrated by imaging the partial back reflection of the trapping beam as it was steered across a stage micrometer. QPD signals were calibrated by transversely sweeping a laser-trapped 2 µm bead through the focus of the 785 nm detection beam in a stepwise manner (5 nm per step). From calibration experiments it was determined that QPD signals were linear with respect to bead displacement if displacements were less than 150 nm. Optical trap stiffness was 30.3±0.5 pN/µm as determined by the power spectrum method as previously described [32].

### Active microrheology analysis

The forcing function acting on a bead was calculated from calibrated laser and bead position signals as described by Brau *et al.* [33]. The laser trap position function given by
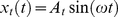
(1)is obtained from the Fourier transform of the calibrated PSD signal, where 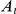 is the amplitude of the transform at the forcing frequency ω. The bead oscillation function given by
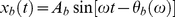
(2)is obtained from the Fourier transform of the calibrated QPD signal, where 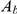 is the bead oscillation amplitude and 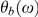 is the phase lag induced by material resistance. The forcing function acting on the bead is calculated from the difference between 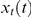 and 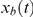


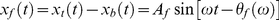
(3)where 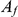 is the amplitude of the forcing function and 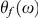 is the phase lag between 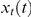 and 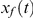. As previous described by Mizuno *et al.* [34], the apparent complex response function 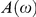,which includes the contribution from trapping forces is
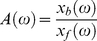
(4)where 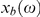 and 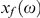 are the Fourier transforms of 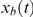 and 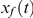 respectively. If the total laser trap contribution 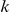 is
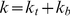
(5)where 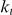 is the stiffness of the trapping beam, and 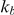 is the stiffness of the detection beam, then the corrected response function 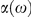 is
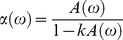
(6), which effectively removes the contribution of optical forces from the measured material properties. Thus, the complex shear modulus G(ω) given by
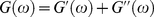
(7)can be calculated from 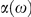 by
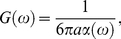
(8)where ‘*a*’ is the radius of the bead. An oscillating bead does work on the local matrix (Fig. 1B), which can either elastically store energy or dissipate it through viscous losses. The elastic and viscous nature of the matrix surrounding a bead is represented by the real and imaginary components of 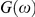 respectively. We validate system performance prior to each experiment by measuring the shear modulus spectra of water (Fig. 1C).

### Smooth muscle cell culture in 3-D fibrin hydrogels

Primary human aortic smooth muscle cells (AoSMC, Lonza) were cultured in SmBM Basal Media (Lonza) supplemented with a SmGM-2 BulletKit (Lonza) at 37°C and 5% CO_2_. The BulletKit contains 5% (v/v) fetal bovine serum, 0.2% (v/v) human basic fibroblast growth factor, 0.1% (v/v) insulin, gentomycin/amphotericin, and human epidermal growth factor. AoSMC-seeded fibrin gels for microrheology experiments were constructed by first dissolving fibrinogen in FBS-free media and then adding cells at 50,000 cells per 1 ml. Gelation was initiated as described above and cells between passages 5 and 7 were used for all experiments. For strain gradient device experiments, cells were cultured in 2.5 mg/ml fibrin gels at 500,000 cells/ml in media supplemented with epsilon-amino-N-caproic acid (e-ACA) at 3 mM to inhibit plasmin mediated fibrin gel degradation [35]. Strain gradients were induced on day 2 and on day 9 gels were formalin fixed and stained for F-actin with Alexa-488 phalloidin per the manufacturer's protocol (Invitrogen).

### Strain gradient device

**Figure 2:**
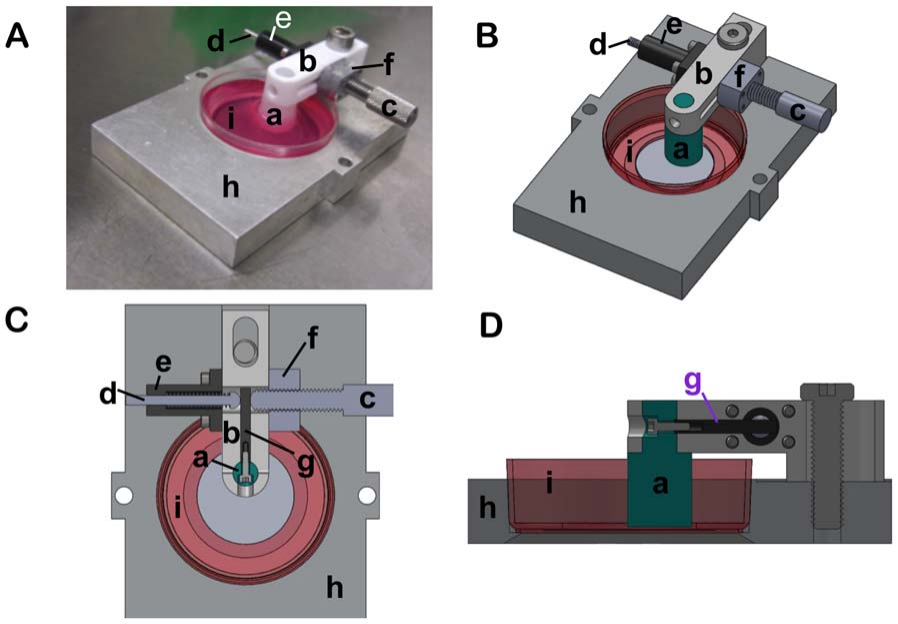
Shear gradient device. (A) Photograph of the assembled device containing a fibrin gel and media containing phenol red. CAD drawings showing: (B) trimetric view of the assembled device, (C) transverse section through a plane dissecting the lever arm, (D) vertical section through the center of the post. The device comprises a post (a) held by a cantilever arm (b), which houses a leadscrew (c) and spring-plunger assembly (d,e) held rigidly by a lead screw block (f). As the thumbscrew is rotated inwards, it pushes the lever arm (g) causing a counter clockwise rotation of the post as viewed from above. As the thumbscrew is rotate out, the spring-plunger assembly pushes the lever arm in the opposite direction causing clockwise rotation of the post. A stage insert (h) houses a Petri dish (i) containing the ECM.

A novel device capable of applying non-uniform strain gradients in a gel polymerized within a 35 mm glass-bottom Petri dish was designed by our group and machined in a facility specializing in medical devices (R.L. Bennett Engineering). The dish is held in a custom-designed stage insert, which also serves as an anchoring point for a cantilever arm housing a leadscrew, spring plunger assembly, lever arm, and rotating post (Fig. 2). The position of the cantilever arm and post is variable in one dimension from the center of the dish to the edge of the imaging area. Translational motion of the leadscrew pushes the lever arm, which is directly coupled to the post and resisted by the spring plunger assembly. After a gel is polymerized in the dish and around the post, rotation of the post induces a strain gradient within the gel. A drawing package is provided online (File S1).

### Finite element model of shear strain

A Finite Elements Analysis (FEA) was performed with the commercial software ABAQUS (Dassault Systèmes Simulia Corp.) to calculate strain profiles along radial and circumferential paths. The modeling space was two-dimensional, with plane stress conditions. The walls of the dish and the plug were each modeled with 200 discrete rigid elements (R2D2), whereas the gel was modeled with 4600 quadratic solid elements, with reduced integration (CPS8R). The gel was tied to both boundaries and assumed to behave as a hyperelastic, neo-hookean solid, with a Poisson's ratio of 0.8, and a true stress – true strain curve in agreement with Janmey *et al* [31]. The dish wall was fixed and the plug was rotated 2.4° counterclockwise about its center point, while being constrained in the radial direction.

### Scanning confocal microscopy

Scanning confocal microscopy was performed using a fluoView1000 (Olympus USA) microscope equipped with a 10× air objective and a 60×, 1.2 NA UPLSAPO water immersion objective (Olympus USA). Samples were excited by a 488 nm Argon laser (Melles Griot) and imaged using standard FITC filters.

### Fibrin mesh analysis

Pore size distributions of fluorescently labeled fibrin gels were obtained from confocal image stacks using a 100 nm step size. Confocal image stacks were reconstructed and segmented in 3D using Volocity (Perkin Elmer), a volumetric analysis software package. The software identifies all touching objects (fibers) within a user defined 3D region of interest (ROI) and segments discrete yet adjacent pores. We applied a lower volume threshold of 0.01 µm^3^. Three ROIs containing approximately 500 pores were analyzed per gel.

### 3D fiber imaging and tracking system

All 3D fibrin fiber imaging and tracking experiments were performed on a custom built two-photon microscope based on an inverted IX70 Olympus microscope, similar to the one described previously [36]. For excitation, we used a mode-locked 80 MHz Ti: Sapphire laser (Chameleon Ultra, Coherent Inc.) with an integrated Verdi pump source. Laser pulses were 150 fs (FWHM) in width, laser average intensity was approximately 150 µW at the sample position, and excitation wavelength was 790 nm for all the tracking experiments. We used an UPlanFL N 60× 0.9 NA air objective (Olympus USA) and a short-pass dichroic mirror (700DCSPXR, Chroma Technology) to direct the excitation light into the sample. Additionally a HQ700LP filter (Chroma Technology) was positioned before the dichroic to filter out the Ti:Sapphire fluorescence. Three-dimensional scanning was obtained using galvanometer motor-driven scanning mirrors (6350, Cambridge Technology Inc.) with controller series 603× servo system (60335 FM, Cambridge Technology Inc.), and a PIFOC P-721 piezo-driven objective device (Physik Instrumente). Both the galvanometer and piezo were driven by an ISS 3-axis card (ISS). Fluorescence signal from the fibers was detected with a photomultiplier tube (H7422P-40, Hamamatsu) through an emission filter (ET680SP, Chroma Technology). Finally, signal was amplified (ACA-4-35N, Becker&Hickl), discriminated (6915, Phillips Scientific, NJ), and TTL pulses were counted by the ISS 3-axis data acquisition card. Experiments were controlled by a commercially available data acquisition program (SimFCS).

### Orbital tracking method

The orbital tracking method has been previously described [37]. Briefly, during each cycle of the tracking routine, the excitation beam traces a circular orbit in a given position around the fiber. The orbit's radius is equal to half the waist of the microscope point spread function (PSF). In the experiments reported in this work, each orbit is in the x-z plane and takes 8 ms. The acquisition rate is chosen such that 128 points are measured during each orbit. After each cycle of the tracking routine, the DC value, AC value, and phase of the first harmonic are calculated on-the-fly by the Fast Fourier Transform (FFT). The modulation (defined as the ratio AC/DC) varies monotonically as the distance from the fiber to the center of the orbit is increased. For every measured value of modulation, we can calculate the distance of the fiber's center of mass from the center of the orbit to determine the coordinates of the fiber. The center of the orbit is relocated to the calculated center of mass. In other words, during the tracking routine the scanner follows the fiber's center of mass by changing its position to that calculated in the previous cycle. When the fiber position is determined with respect the x-z plane, the orbit is moved according to a linear ramp function incrementing the orbit position in the y-direction along the fiber and a new cycle of the tracking routine starts. Given the highly branched nature of the fibrin network, it is possible that tracking can erroneously deviate from one fiber to a neighboring fiber. As a control, we analyze orbit coordinates and fluorescence intensity as a function of time after tracking is complete. If the tracking jumps to a neighboring fiber, there will be an abrupt change in the modulation of the first harmonic and a spike in the intensity. Fibers were tracked a maximum distance of 10 µm over 120 seconds with a step size resolution of 56 nm. All measured fibers were located at least 3 µm above the cover slip to avoid surface effects. The y-position of the orbit was sequentially moved through approximately 1500 positions along each fiber. For display, the color of the reconstructed trajectory changes every 100 measured points.

### Statistical analysis

All data are expressed as mean and standard deviation. The Student's t-test was used to test differences between means with a level of significance of 0.05, unless otherwise indicated.

## Results

### AMR reveals mechanical heterogeneity of fibrin gels

**Figure 3:**
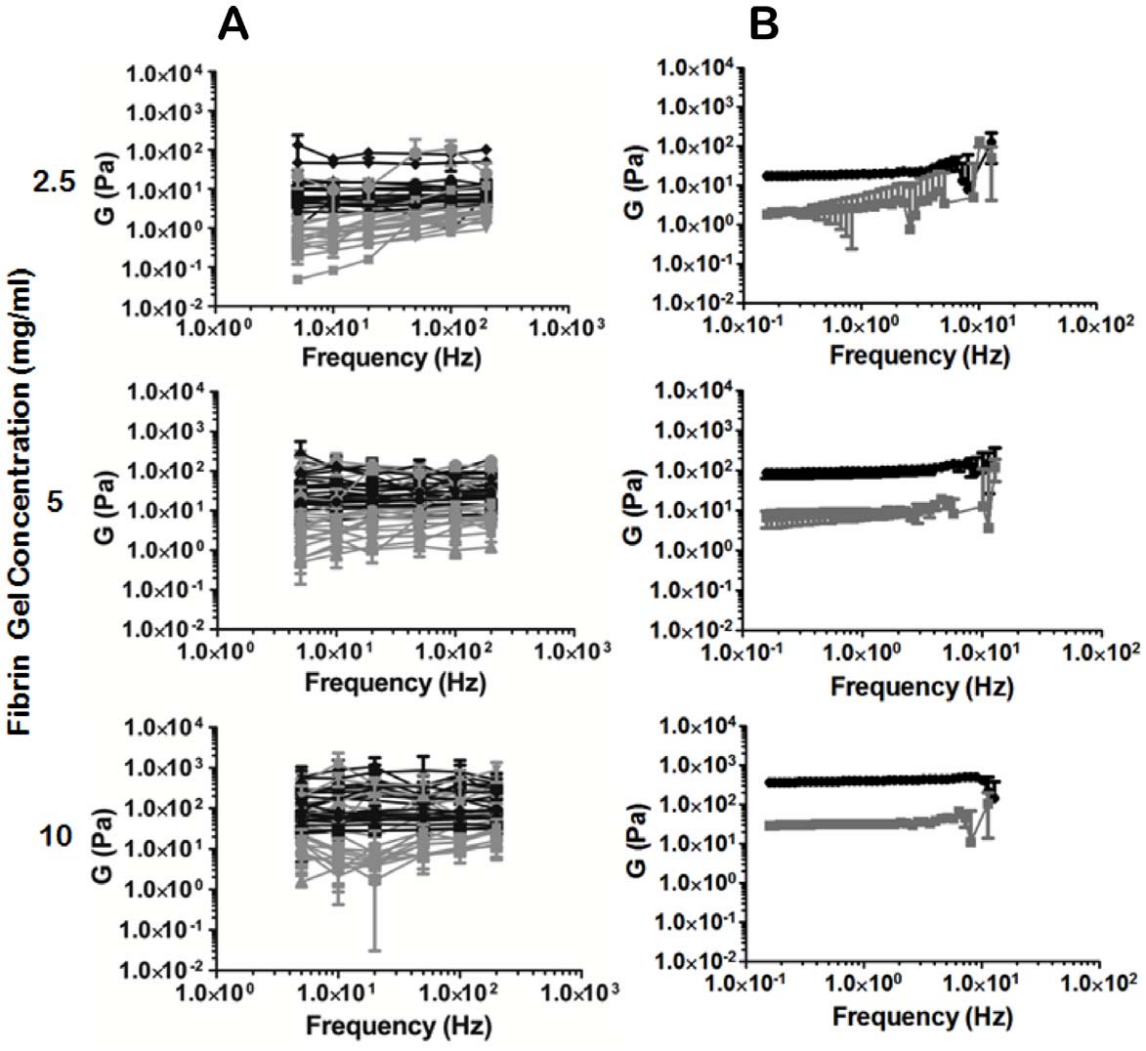
Microrheology reveals heterogeneity unresolved by parallel plate rheology. Fibrin gels polymerized at 2.5, 5 and 10 mg/ml fibrin were measured by both AMR (A) and parallel plate rheology (B). Each curve in (A) represents AMR for a different bead measured at 5, 10, 20, 50, 100 and 200 Hz (black = G′, grey = G″). Errorbars represent the standard deviation of G for five sequential measurements. Errorbars in (B) represent the standard deviation in G measured for five gels. AMR measured for the three concentrations of fibrin demonstrate considerable overlap as compared to parallel plate rheology.

Both AMR and parallel plate macrorheology were used to measure complex shear moduli in fibrin gels polymerized from 2.5, 5, and 10 mg/ml solutions of fibrinogen. AMR of fibrin gels measures a large variation in stiffness between microdomains in a single gel (Fig. 3A), while macrorheology measures an ensemble average, which is insensitive to the distribution in microdomain stiffness (Fig. 3B). Therefore, parallel plate rheology fails to report local heterogeneities in gel stiffness detectable by AMR. We found agreement between macro and microrheology of 2.5 mg/ml fibrin gels, but not for 5 mg/ml and 10 mg/ml fibrin, where microrheology reports a softer matrix than macrorheology (Table 1). Both rheology techniques report elastic shear moduli increasing linearly with fibrin concentration (macro: r^2^ = 0.98, micro: r^2^ = 0.97), although nonlinearities would be observed at higher strains.

**Table 1:** Comparison of fibrin parallel plate and microrheology.

[Fibrin] (mg/ml)	G′ macro (Pa)	G′ AMR (Pa)	p-value
2.5	18±2.5	13±20	0.45
5	90±7.6	43±35	<0.05
10	377±30	186±182	<0.05

**Figure 4:**
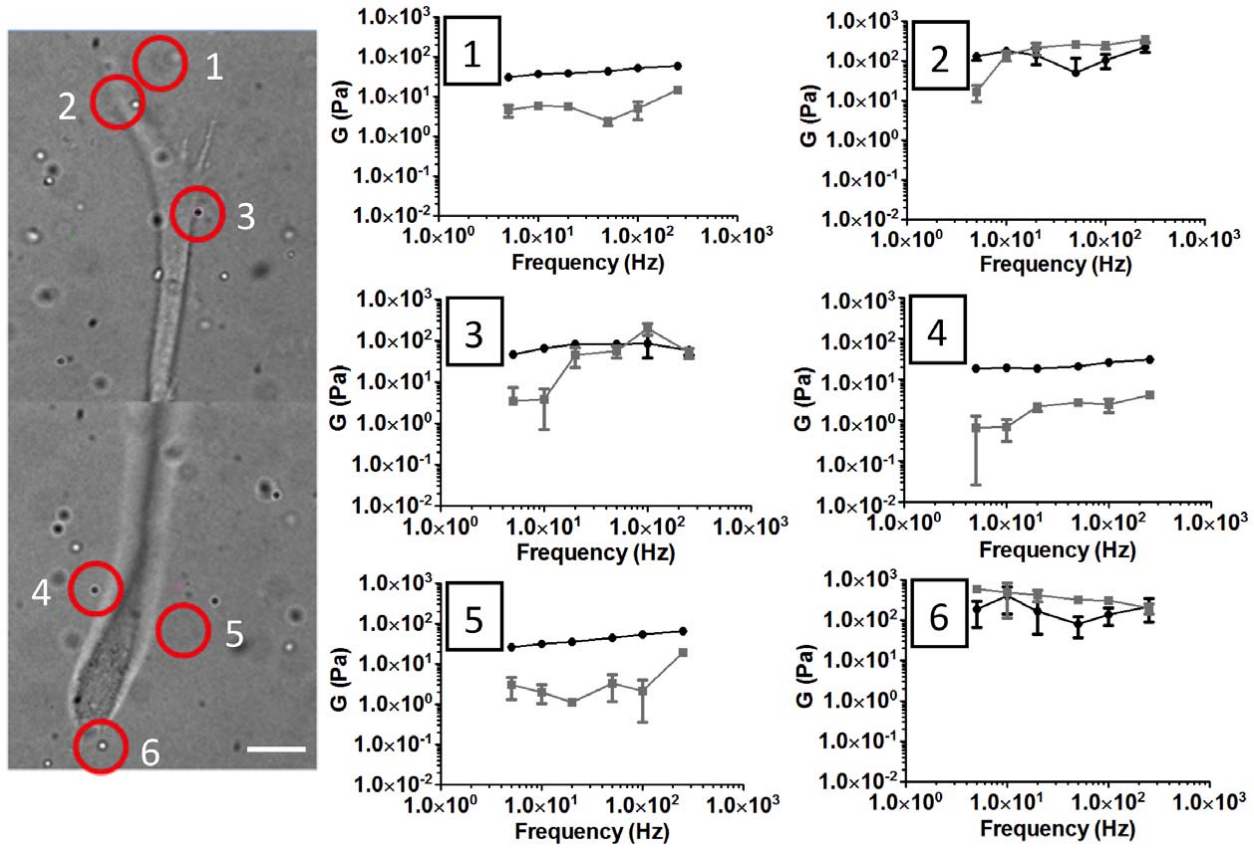
Microrheology reports local mechanical heterogeneity around a single cell. An AoSMC cultured in a 3D fibrin gel polymerized at 2.5 mg/ml fibrin. Six beads proximal to the cell were chosen at random for AMR. Note the 10-fold difference in G′ between beads 4 and 6. Black = G′, grey = G″, scale bar = 20 µm.

The importance of measuring local stiffness is highlighted in Fig. 4, in which the material stiffness varies by a factor of 10 around the periphery of a single AoSMC cultured in a 3D fibrin gel, an observation not detectable by macrorheology. Furthermore, regions measured at the polar contracting ends of the cell (beads 2 and 6) are noticeably stiffer than those regions located along the length of its body (beads 3–5).

### AMR in the strain gradient device: spatially dependent stiffening

**Figure 5:**
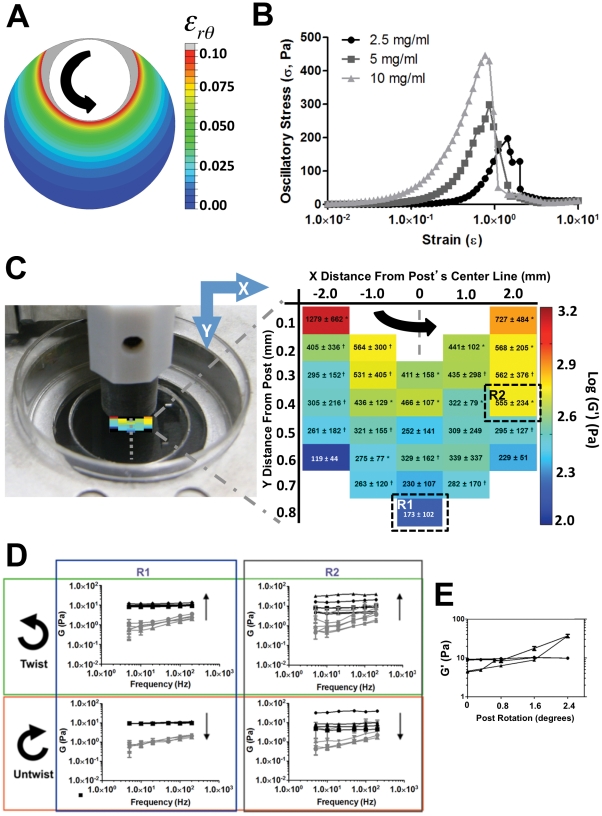
Microrheology within the strain gradient device. AMR within a 2.5 mg/ml fibrin gel confirms location-dependent stiffening with rotation of the post. (A) FEA of shear strain, 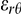, in response to a 2.4° rotation of the post. (B) Stress-strain curves for parallel plate rheology strain sweeps of 2.5, 5, and 10 mg/ml fibrin gels report nonlinear stiffening. (C) AMR map of G′ in a region near the post. In the first 0.1 mm from the post, changes in G′ are largest in the circumferential direction. For each colored block, G′ is compared to that of the unrotated state (*p<0.05; ^†^p<0.15). (D) AMR for a single bead in either region R1 or R2 as specified in (C). AMR was performed as the post was rotated by 0.8, 1.6 and 2.4°, and then again as the post was rotated back. In region R1, no stiffening was observed as compared to R2 where G′ increased with rotation and then decreased as the post was rotated back to its original position. (E) Stiffening in region R2 (triangles) exhibited hysteresis where the material stiffened and softened along different paths as the post rotated. Note that the material returned to its original stiffness as measured by AMR. No change in stiffness was observed in region R1 (circles). Each point is the mean G′ of all measured frequencies. Errorbars represent the standard deviation of 5 sequential measurements.

In our device, local gel stiffness was modulated by rotation of the post as measured *in situ* by AMR. We observed that stiffness could be modulated independent of initial fibrinogen concentration following a 2.4° rotation of the post within a 2.5 mg/ml fibrin gel. For these conditions, FEA estimates a nonuniform distribution of shear strain within the Petri dish (Fig. 5A). Strain is greatest at the gel-post interface, decreasing radially in a steep gradient towards the edge of the dish. The eccentric placement of the post within the dish induces a large variation in strain ranging from 0.005 to 1.3. Parallel plate rheology (constant frequency) reports nonlinear strain stiffening for 2.5, 5 and 10 mg/ml fibrin gels, with the 2.5 mg/ml gels stiffening linearly for strains below 0.1 (Fig. 5B).

To directly measure the heterogeneity in stiffness within our device, we performed AMR at 30 positions throughout a region of the gel as indicated in Fig. 5C. At the center of each region, five neighboring microbeads were probed to determine the local distribution of G′. We observed a pattern of stiffening consistent with our FEA model of strain and macroscopic measurement of strain stiffening. Importantly, we determined that the large endogenous variability in G′ measured in unstressed gels could not account for the observed stiffening in our device, where the level of significance was relaxed to 0.15 for several regions. In further support of strain stiffening, we measured G for a single bead as the post was rotated by 0.0, 0.8, 1.6, and 2.4° in both region R1 and R2 in Fig. 5D. AMR in R1 reported no significant strain stiffening of the matrix even at 2.4° rotation of the post (Fig. 5D), a finding consistent with AFM studies of fibrin stiffness at low strain [31]. AMR in R2 reports both a 10-fold stiffening as well as hysteresis (Fig. 5D, E).

**Figure 6:**
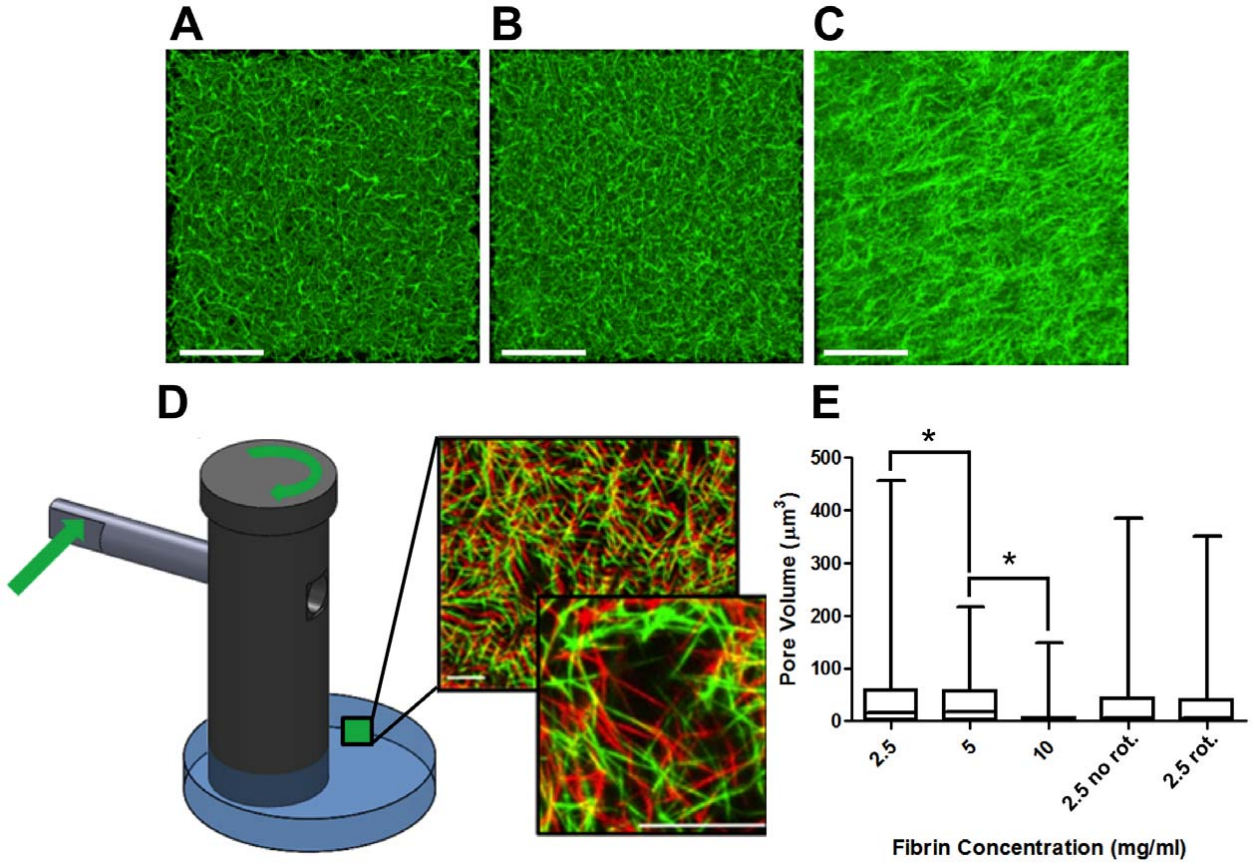
Matrix deformation does not accompany stiffening as observed by confocal microcopy. Maximum projection images computed from confocal stacks of fluorescently labeled fibrin gels polymerized within a Petri dish at 2.5 (A), 5 (B) , or 10 (C) mg/ml show decreasing pore size with increasing concentration. The 10 mg/ml gel exhibits fiber bundling. (D) A 2.5 mg/ml fibrin gel was polymerized within the shear gradient device. After rotation of the post by 2.4° there is a visible displacement in the gel in region R2 (see Fig. 5), with little deformation in matrix geometry. (E) Comparison of fibrin pore volume shows significant pore volume reduction with increasing fibrin concentration, but not with rotation of the post in region R2 of the device, where AMR reports 10-fold stiffening (*p<0.05).

As shown above in Fig. 3A, AMR reports an increase in stiffness with increasing fibrin concentration in unstressed gels. This finding is consistent with the observed increase in mesh density (Fig. 6A, B, C) and formation of fiber bundles (Fig. 6C) by scanning fluorescence confocal microscopy. Surprisingly, examination of the 2.5 mg/ml fibrin mesh in R2 following rotation of the post by 2.4° suggests a translation of the mesh with no significant change in pore geometry for strained gels (Fig. 6D). Measured pore volume decreased with increasing fibrin concentration in unstressed gels (p<0.05), but not in R2 following rotation of the post (p>0.05, Fig. 6E). Thus local stretch can induce 10-fold stiffening without large deformations in pore geometry as assessed by diffraction limited light confocal microscopy.

### Orbital tracking reveals nanostructural changes to fibers

**Figure 7:**
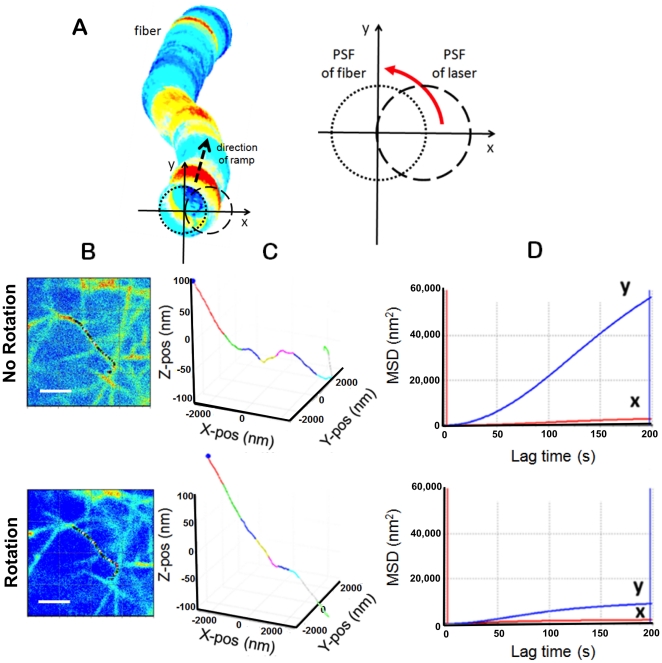
3D multiphoton orbital tracking shows nanostructural fiber changes. (A) In 3D multiphoton orbital tracking the position of multiphoton fluorescence is calculated from intensity modulation of the point spread function as the laser is rapidly scanned in an orbit around the fiber. Fibers are tracked as the orbit is moved in the fiber direction by a linear ramp function. (B) Multiphoton images of a region in a fibrin gels before and after rotating the post by 2.4°. Fibrin was labeled with ANS (scale bar = 20 µm). (C) Tracked fiber before and after rotation of the post shows elongation and straightening. With no rotation the fiber has nanometer-scale structure. (D) MSD for the fiber midpoint in both the y and x directions with and without rotation of the post. Since the fiber runs nearly parallel to the x-axis of the orbital tracking coordinate system, the MSD is relatively flat along that axis, as compared to the y-axis where significant reduction in MSD is seen once the post is rotated.

To further investigate the mechanical basis of stiffening in R2, we implemented orbital tracking of fibers (Fig. 7A) with and without applied stretch. In the absence of stretch, fibers appear buckled and coiled at the nanometer-scale, implying a slack state. Following rotation of the post by 2.4°, individual fibers transition from coiled (Fig. 7B, C, top row) to straightened and elongated (Fig. 7B, C, bottom row) consistent with the conformational change of a rope-like fiber under tension. In support of increased fiber tension with stretch, the maximum value of the MSD of the fiber midpoint after 200 seconds decreased with rotation of the post from approximately 60,000 nm^2^ to 10,000 nm^2^ (Fig. 7D). Moreover, fibers in this region were observed to recover to their original conformation as the post was unrotated (data not shown), consistent with G′ recovery measured by AMR (Fig. 5E).

### Smooth muscle cell culture in the strain gradient device

**Figure 8:**
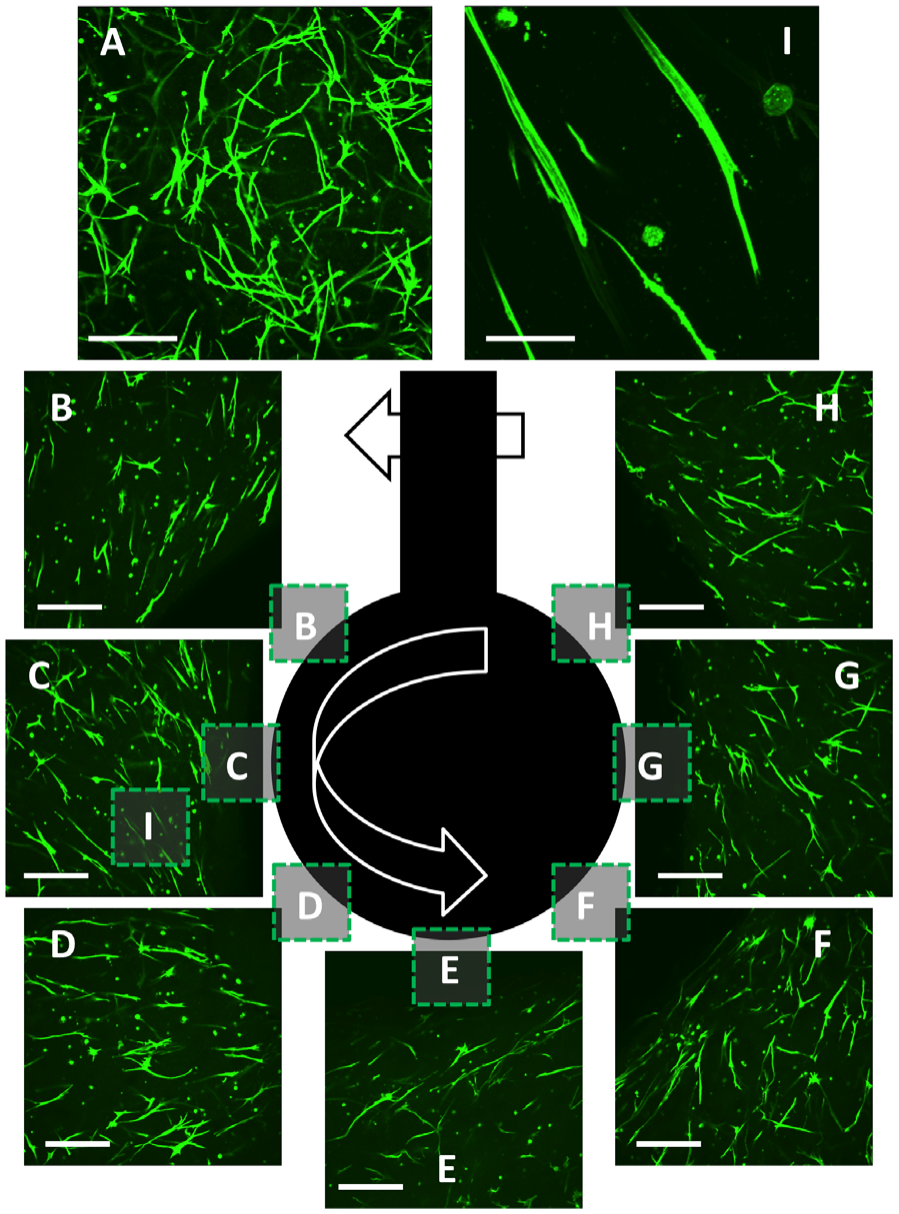
Aortic smooth muscle cells align with stiffness gradient. AoSMCs were cultured in a fibrin gel polymerized at 2.5 mg/ml fibrin and subjected to rotation of the post by 2.4° on day 2. Cells were fixed and labeled with Alexa-488 Phalloidin on day 9 for confocal microscopy with a 10× microscope objective. (A) Far from the post cells are randomly oriented and relatively abundant. (B–H) Cells within several hundred microns from the post align in a direction more circumferential than radial. (I) a region in (C) imaged with a 60× water immersion objective. Scale bar = 300 µm (A–H), scale bar = 50 µm (I).

Cells located far from the post in non-stiffened regions of the device were randomly oriented (Fig. 8A). In contrast cells located near to the post appear partially aligned with the direction of post rotation (Fig. 8B–H). In particular, cells located less than approximately 200 µm from the post surface appear oriented with their long axis more tangent than normal to the surface of the post (Fig. 8B–H). AMR measurements of acellular fibrin gels in Fig. 5C indicate steep circumferential gradients within this region. Cells farther from the post exhibit a random orientation.

## Discussion

We have presented a method for tuning the mechanical properties of naturally derived ECMs. Paramount to our method, we emphasize the need to measure material properties at the same length scale as cells. Typically, material stiffness is measured macroscopically where the material can be assumed to act as a continuum. In order for the continuum assumption to apply, the characteristic length scale of underlying structural components must be much smaller than that of the physical model [26]. In our system, fiber structure and architecture are near to the same scale as the cell itself, as evidenced by comparing the features of fibrin ECMs (Fig. 6A–C) to the size of a single cell in Fig. 4. This compels us to probe the complex heterogeneous fibrous system by quantitative methods, such as AMR, to elucidate the role that local matrix stiffness plays in cellular physiology. Bulk measurements are in general insensitive to local micron scale heterogeneities, shown by the comparison between micro and macro scale rheology of fibrin gels polymerized at 2.5, 5 and 10 mg/ml (Fig. 3). While the increase in stiffness with fibrin concentration was expected, the discrepancy between AMR and macrorheology highlights the requirement of measuring local micromechanics, particularly given the significant overlap in microrheological stiffness of 2.5, 5 and 10 mg/ml fibrin gels.

The spatial variability in stiffness revealed by AMR is expected under the assumption that local viscous and elastic moduli are dependent upon local mesh geometry, which is notably heterogeneous (Fig. 6A). In addition, the stiffness of an ECM is subject to temporal variability as cells dynamically alter their microenvironment through remodeling and the generation of cytoskeletal traction forces. While it is likely that cells actively remodel the local matrix through protease activity [38] and the deposition of new ECM [39], it is also known that fibrin stiffness is modulated by cell-mediated mechanical stress as measured by AFM [31]. This is because fibrin gels exhibit strain stiffening, which is typical of soft biological materials [15]. It is important to note that methods of 3D traction force microscopy [40] applied to naturally derived materials [30] must include *in situ* real time measures of local stiffness since there will not be a simple relationship between bead displacement and cell forces when material properties are spatially and temporally variant. AMR's ability to track changes in local stiffness, in real time, make it an appropriate technique to complement 3D traction force microscopy.

As implemented here, AMR measures ECM stiffness in a local volume just larger than the bead. As a result, AMR has micron resolution allowing spatial mapping of stiffness around a cell as it dynamically interacts with its ECM as shown in Figure 4. Here, the measured distribution of elastic moduli surrounding the cell implies it is exerting traction forces at its polar ends, which would cause local stretch-induced stiffening, consistent with observations of bead displacement for cells migrating in 3D [30]. Therefore, we assert that in order to generate a complete model of the role of ECM stiffness in cell regulation, we must map both the endogenous stiffness as well as local cell-mediated mechanical changes using a nondestructive method such as AMR.

Our novel cell culture device enables users to tune the stiffness of naturally derived ECMs, while allowing for continuous optical observation and interrogation. We demonstrated that within a single 35 mm culture dish we could tune stiffness independent from matrix architecture in a naturally derived matrix such as fibrin. Local gel mechanics were altered, not through increased protein concentration or exogenous cross-linking, but by directly altering the tensile stress state of the gel's fiber network through the application of shear strain. In contrast to polymer-peptide hybrid systems, our device allows cell-cell communication of soluble factors through ‘natural’ pores, where interacting cells may independently experience very different mechanical microenvironments. As predicted by FEA, we observed a strong circumferential gradient in stiffness near to the post as well as regions far from the post in which the distribution of stiffness was unaltered. While the model in conjunction with parallel plate rheology predicts a smooth distribution in stiffness, AMR reports a noisy distribution (Fig. 5C) due to the large endogenous heterogeneity in mesh architecture. Furthermore, the differential stiffening observed in regions R1 and R2 (Fig. 5D) at varying degrees of rotation of the post highlight our ability to tune local mechanics by the application of strain. In high strain regions, we measured hysteresis in stiffness, where the original stiffness was entirely recoverable. This result is consistent with observations of fibrin's mechanical integrity under large strains [15].

Surprisingly, we achieved a ten-fold stiffening within a 2.5 mg/ml fibrin gel as measured by AMR with no detectable change in pore geometry as measured by fluorescence confocal microscopy (Fig. 6D) and image analysis of pore volume (Fig. 6E). While we did not observe the expected collapse of pores or significant fiber alignment with the direction of stretch [15], we did detect straightening and elongation of fibers at the nanoscale by orbital tracking (Fig. 7). These nanostructural and mechanical changes measured in fibrin are consistent with recent multiscale theories claiming that fibrin's resiliency arises from molecular level extension and alignment [15], [17]. A plausible explanation of the observed stiffening is that the strain induced by rotation of the post engaged covalent bonds within fibrin fibers thus stiffening the matrix as reported by AMR.

We observed differential AoSMCs alignment within our device suggesting cells were sensitive to the induced stiffness gradient. AoSMCs showed a strong circumferential alignment in the first few hundred microns from the post's surface. This is in agreement with FEA and AMR, which predicts circumferentially aligned principle strain, and reports strong circumferential gradients in stiffness respectively. Cells further from the post were randomly oriented, as were cells cultured in control dishes (data not shown). The random orientation of cells in these low strain regions is consistent with AMR results (Fig. 5C), which indicate that the distribution of stiffness was unaltered with the application of strain. This preliminary result suggests that cells can respond to stiffness gradients developed in naturally derived ECMs by the application of non-uniform stretch. Here we have demonstrated both the ability to measure and tune stiffness as well as the ability to image cell responses by diffraction limited fluorescence microscopy.

In the context of available photonic technologies such as microrheology, confocal imaging, and multiphoton 3D particle tracking, as well as techniques not yet realized, this device provides an appropriate platform in which to study cell-ECM interactions while manipulating local mechanics of naturally derived ECMs without altering their composition. In this paper we have demonstrated the utility of this device in the context of fibrin, however, our device can be applied to many ECMs already in use by cell biologists such as those derived from collagen and agarose as well as commercial products such as Matrigel and custom designed protein-polymer hybrids.

## Supporting Information

File S1 **Drawing package of shear gradient device.** The drawing package contains part-by-part drawings as well as assembly diagrams. The drawing packing is complete and can be submitted as is to a qualified machine shop.
(PDF)
