# Supplementary material for: Concentration Independent Modulation of Local Micromechanics in a Fibrin Gel
Source: PLoS One. 2011 May 23;6(5):e20201. doi: 10.1371/journal.pone.0020201 (PMC3100350; doi:10.1371/journal.pone.0020201)
Supplement: File S1 — Drawing package of shear gradient device. The drawing package contains part-by-part drawings as well as assembly diagrams. The drawing packing is complete and can be submitted as is to a qualified machine shop. (PDF) [file pone.0020201.s001.pdf]

GENERAL NOTES:

REVISION	MODELED BY	DRAWN BY	CHECKED BY	DATE
A	SAMIR SHREIM	SAMIR SHREIM	SAMIR SHREIM	5/21/2009
B	SAMIR SHREIM	SAMIR SHREIM		

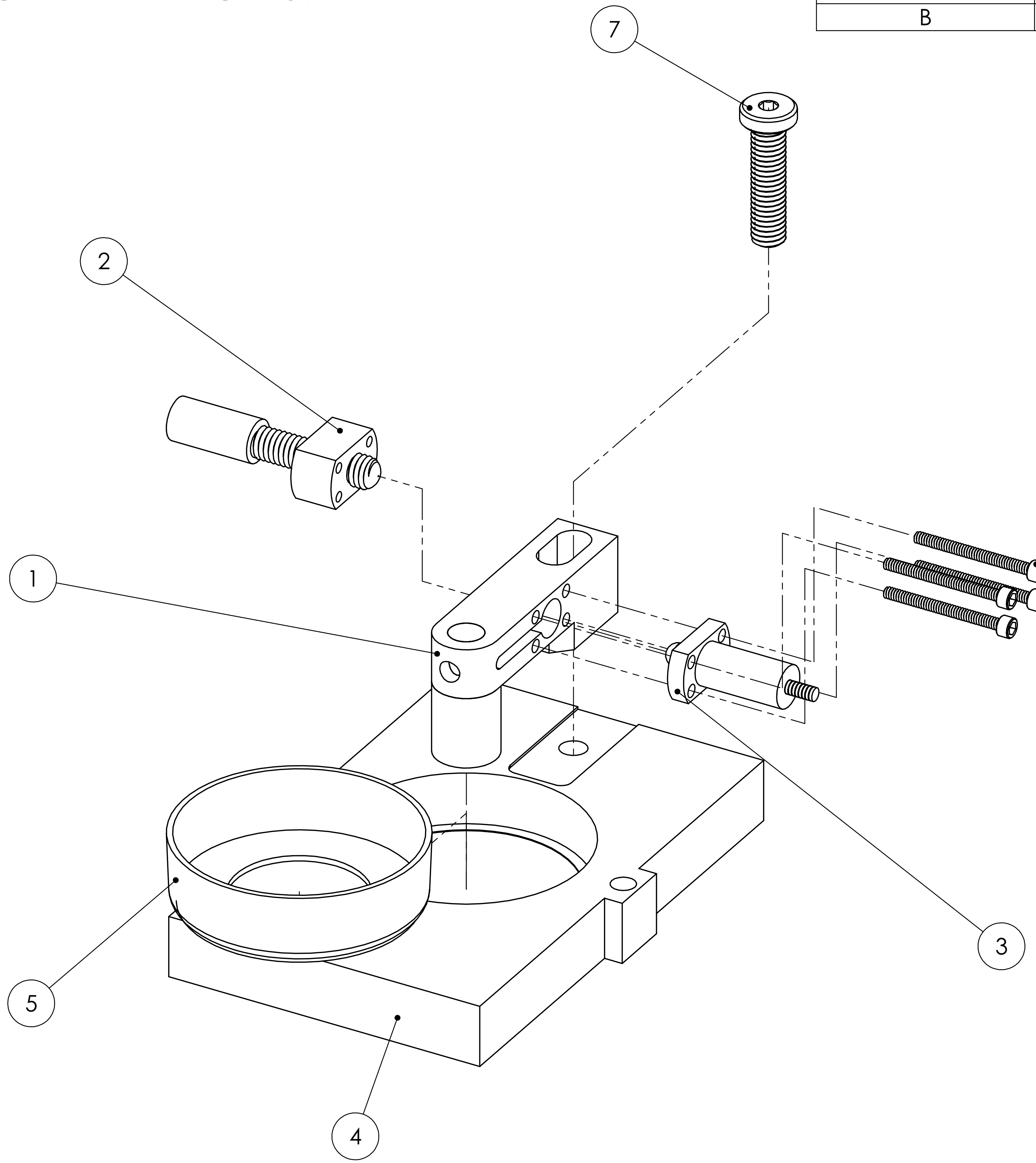

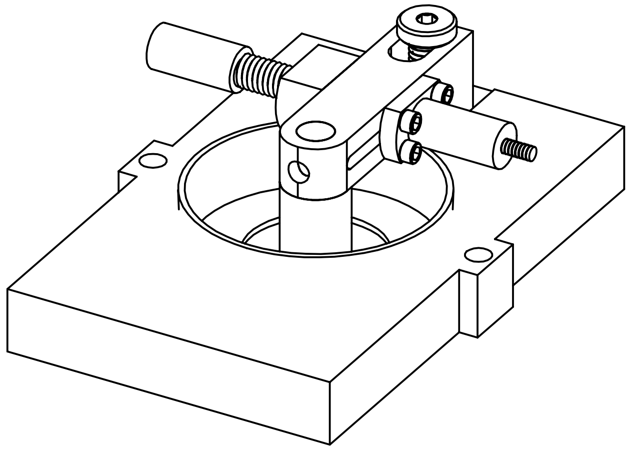

ITEM NO.	PART NUMBER	DESCRIPTION	QTY.
1	107-001-000	CROSS ARM ASSEMBLY	1
2	107-002-000	LEADSCREW ASSEMBLY	1
3	107-003-000	SPRING PLUNGER ASSEMBLY	1
4	107-000-001	BASEPLATE	1
5	-	MATTEK P35G-1.5-20-C GLASS BOTTOM CULTURE DISH	1
6	-	1-64 X 0.725 SHCS, BRIGHTON BEST 592060	4
7	-	10-32 X 3/4 LOW PROFILE SHCS, MCMASTER CARR 93615A385	1

UNLESS OTHERWISE SPECIFIED
DIMENSIONS ARE IN INCHES
TOLERANCES:
FRACTIONAL ± 1/32
ANGULAR: MACH ± 0.5 DEG
TWO PLACE DECIMAL ±0.01
THREE PLACE DECIMAL ±0.005

TITLE:
SHEAR GRADIENT DEVICE

PROPRIETARY AND CONFIDENTIAL

THE INFORMATION CONTAINED IN THIS DRAWING IS THE SOLE PROPERTY OF THE BEAMS LAB at UCI'S BECKMAN LASER INSTITUTE. ANY REPRODUCTION IN PART OR AS A WHOLE WITHOUT THE WRITTEN PERMISSION OF THE BEAMS LAB IS PROHIBITED.

SIZE	DWG. NO.	REV
B	107-000-000	B

SCALE: 1.5:1 WEIGHT: SHEET 1 OF 1

REVISION	MODELED BY	DRAWN BY	CHECKED BY	DATE
A	SAMIR SHREIM	SAMIR SHREIM	SAMIR SHREIM	5/21/2009
B	SAMIR SHREIM	SAMIR SHREIM		

GENERAL NOTES:

1. MATERIAL: 6061 ALUMINUM

2. FRICTION FIT WITH MATTEK P35G-1.5-20-C (35 mm GLASS BOTTOM CULTURE DISH)
DISH SHALL NOT ROTATE UNDER FULL RANGE OF ROTATOR SHAFT ACTUATION

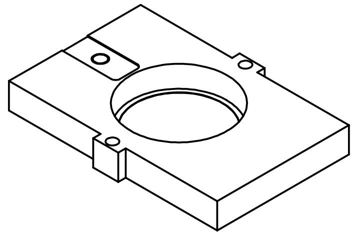

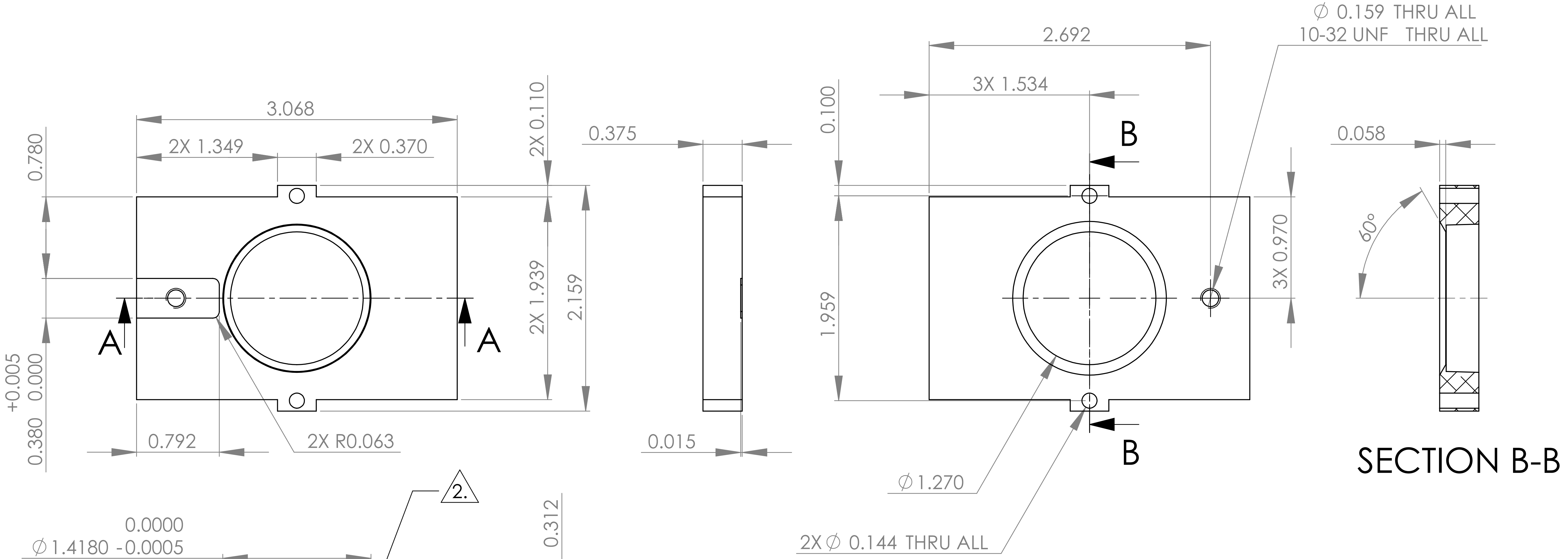

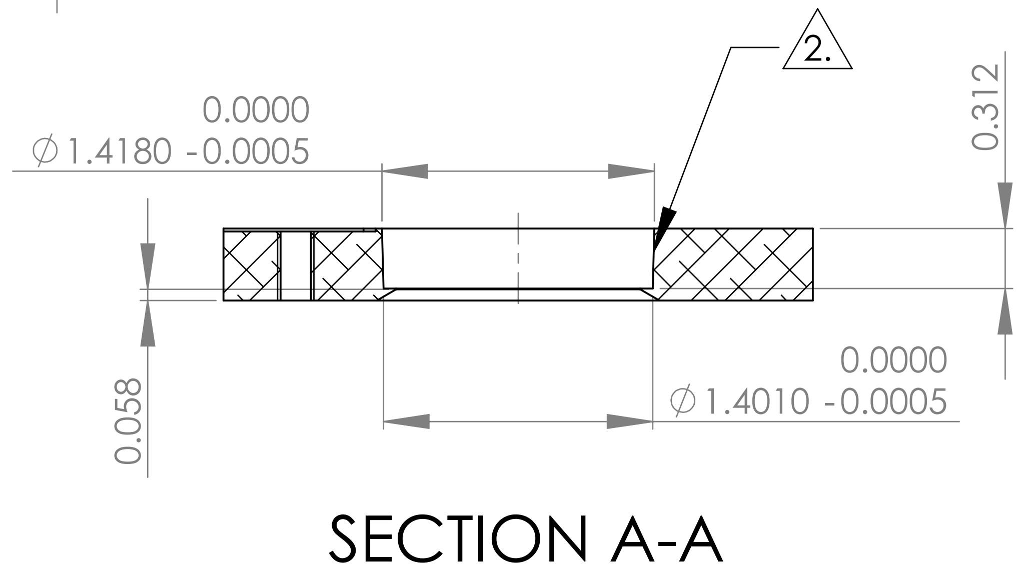

UNLESS OTHERWISE SPECIFIED
DIMENSIONS ARE IN INCHES
TOLERANCES:
FRACTIONAL ± 1/32
ANGULAR: MACH ± 0.5 DEG
TWO PLACE DECIMAL ±0.01
THREE PLACE DECIMAL ±0.005

TITLE: BASEPLATE

PROPRIETARY AND CONFIDENTIAL
THE INFORMATION CONTAINED IN THIS DRAWING IS THE SOLE PROPERTY OF THE BEAMS LAB at UCI'S BECKMAN LASER INSTITUTE. ANY REPRODUCTION IN PART OR AS A WHOLE WITHOUT THE WRITTEN PERMISSION OF THE BEAMS LAB IS PROHIBITED.

SIZE	DWG. NO.	REV
B	107-000-001	B

SCALE: 1:1 WEIGHT: SHEET 1 OF 1

REVISION	MODELED BY	DRAWN BY	CHECKED BY	DATE
A	SAMIR SHREIM	SAMIR SHREIM	SAMIR SHREIM	5/21/2009
B	SAMIR SHREIM	SAMIR SHREIM		

GENERAL NOTES:

1. BUSHING FIT AT 37°C 100% HUMIDITY

2. BUSHING FIT AT 37°C 100% HUMIDITY

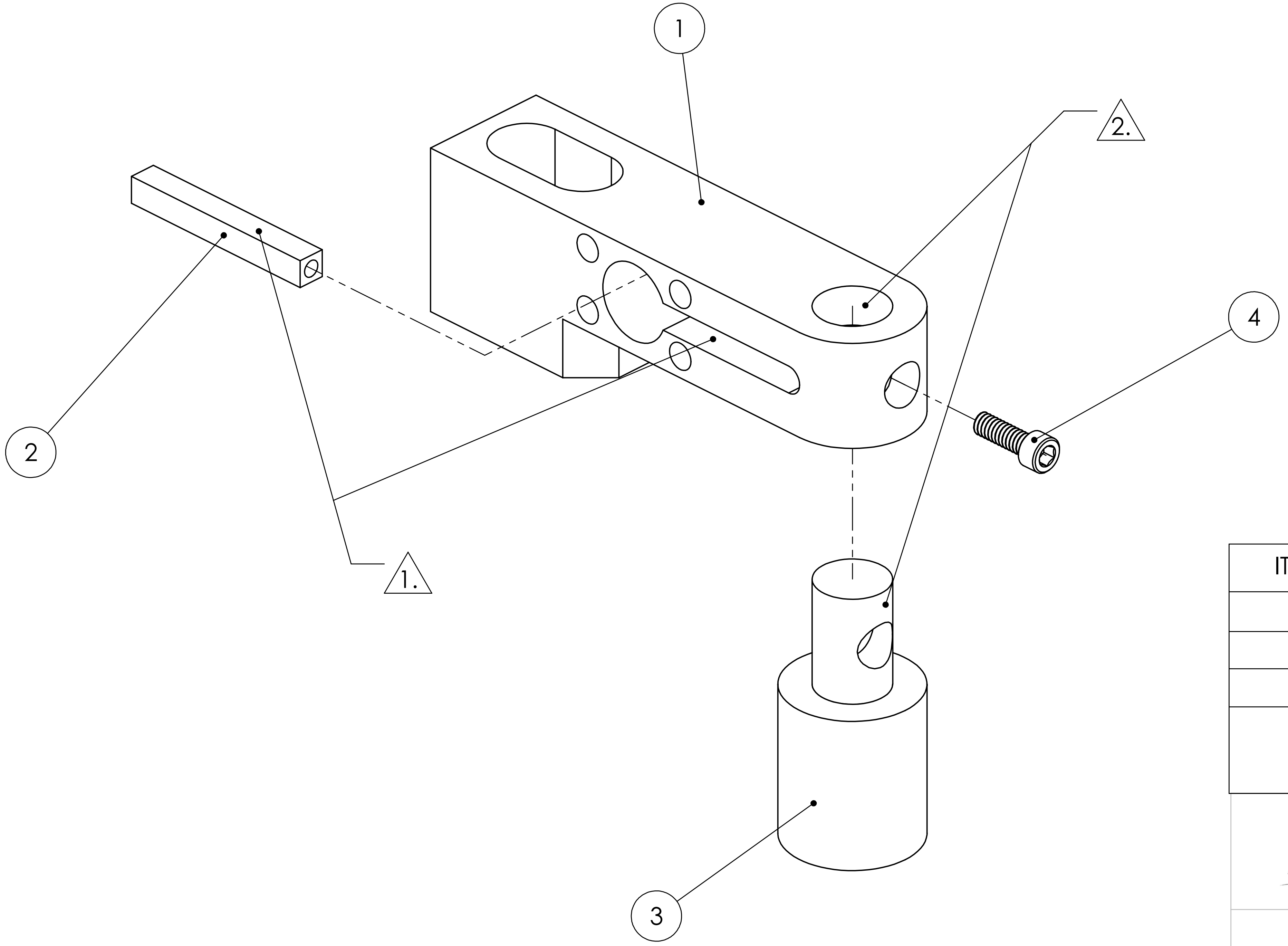

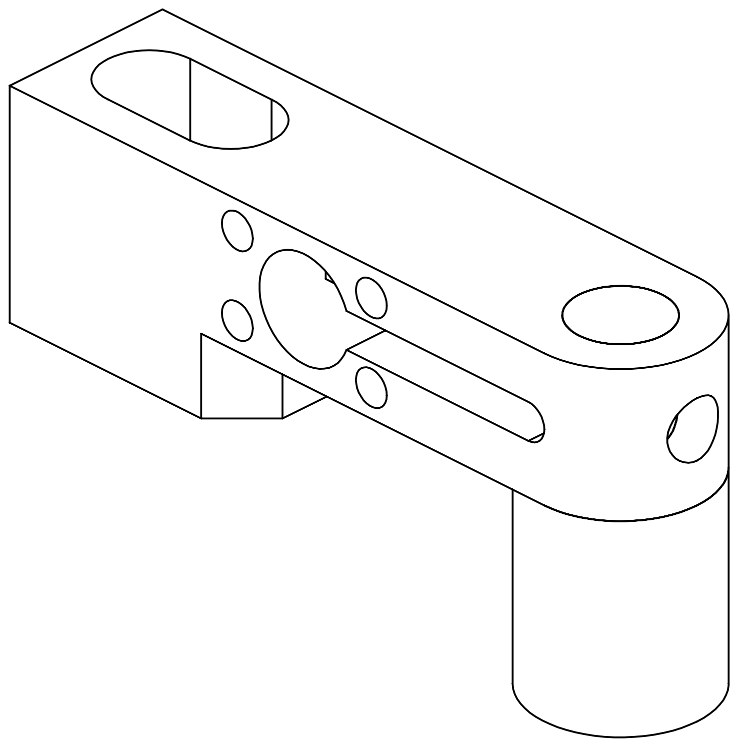

ITEM NO.	PART NUMBER	DESCRIPTION	Exploded View/QTY.
1	107-001-001	CROSS ARM	1
2	107-001-002	LEVER ARM	1
3	107-001-003	ROTATOR SHAFT	1
4	-	0-80 X 3/16 SHCS STAINLESS, MCMASTER CARR P/N: 92196A054	1

UNLESS OTHERWISE SPECIFIED
DIMENSIONS ARE IN INCHES
TOLERANCES:
FRACTIONAL ±1/32
ANGULAR: MACH ± 0.5 DEG
TWO PLACE DECIMAL ±0.01
THREE PLACE DECIMAL ±0.005

TITLE:
CROSS ARM ASSEMBLY

PROPRIETARY AND CONFIDENTIAL

THE INFORMATION CONTAINED IN THIS DRAWING IS THE SOLE PROPERTY OF THE BEAMS LAB at UCI'S BECKMAN LASER INSTITUTE. ANY REPRODUCTION IN PART OR AS A WHOLE WITHOUT THE WRITTEN PERMISSION OF THE BEAMS LAB IS PROHIBITED.

SIZE B | DWG. NO. 107-001-000 | REV B

SCALE: 3:1 | WEIGHT: | SHEET 1 OF 1

GENERAL NOTES:

REVISION	MODELED BY	DRAWN BY	CHECKED BY	DATE
A	SAMIR SHREIM	SAMIR SHREIM	SAMIR SHREIM	5/21/2009
B	SAMIR SHREIM	SAMIR SHREIM		

1. MATERIAL: TEFLON (PTFE)

2. BUSHING FIT WITH ROTATOR SHAFT AT 37°C 100% HUMIDITY

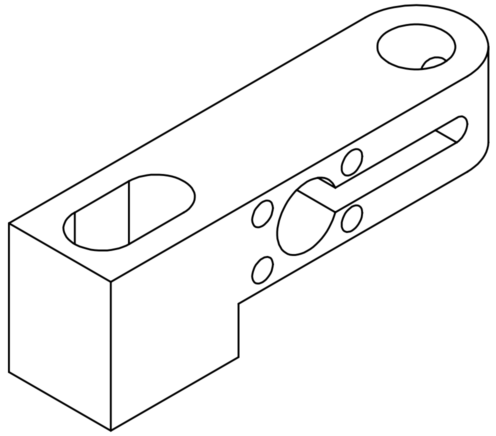

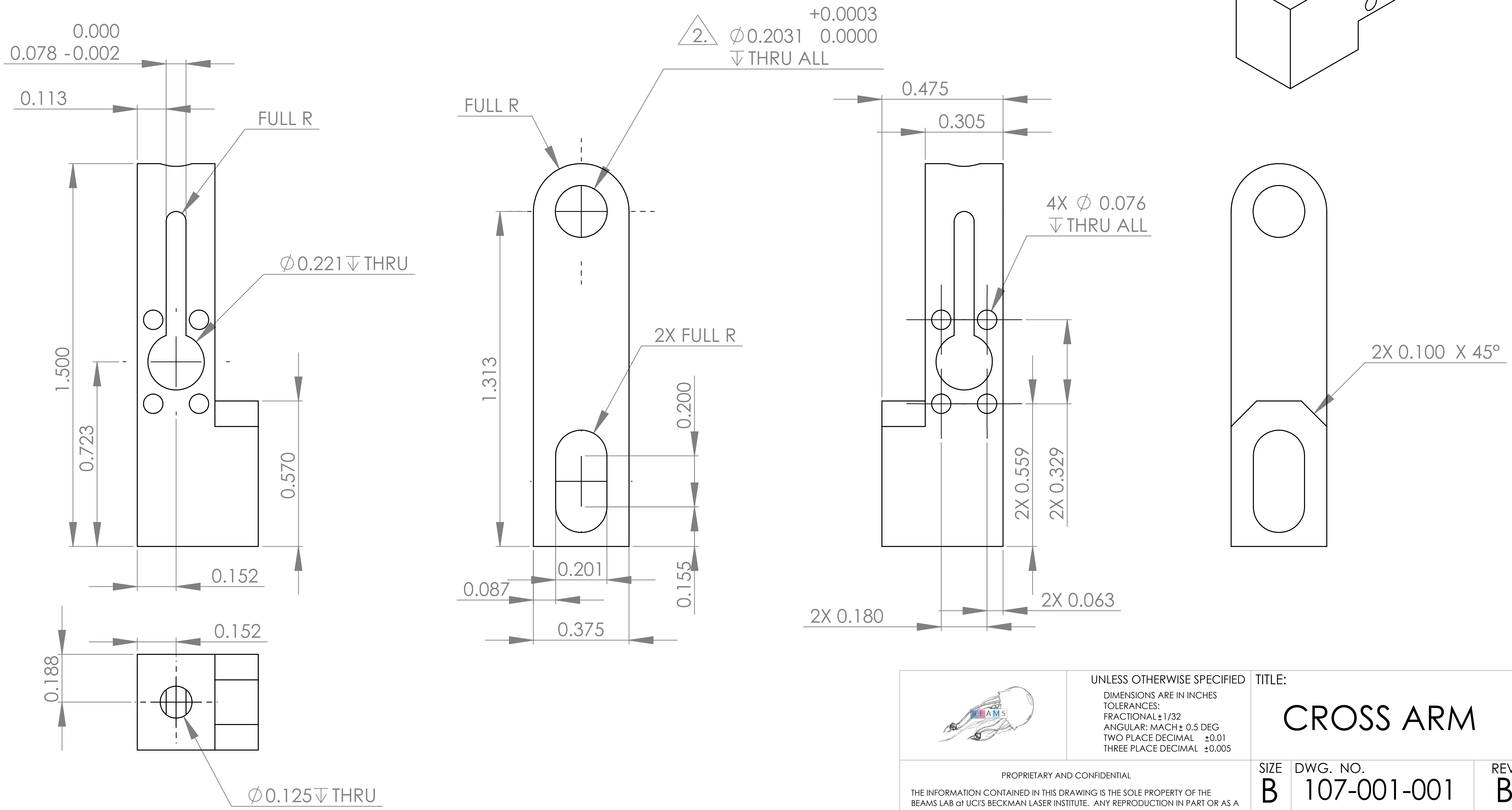

UNLESS OTHERWISE SPECIFIED
DIMENSIONS ARE IN INCHES
TOLERANCES:
FRACTIONAL ± 1/32
ANGULAR: MACH ± 0.5 DEG
TWO PLACE DECIMAL ±0.01
THREE PLACE DECIMAL ±0.005

TITLE:

CROSS ARM

SIZE: B | DWG. NO.: 107-001-001 | REV: B

SCALE: 2.5:1 | WEIGHT: | SHEET 1 OF 1

PROPRIETARY AND CONFIDENTIAL

THE INFORMATION CONTAINED IN THIS DRAWING IS THE SOLE PROPERTY OF THE BEAMS LAB at UCI'S BECKMAN LASER INSTITUTE. ANY REPRODUCTION IN PART OR AS A WHOLE WITHOUT THE WRITTEN PERMISSION OF THE BEAMS LAB IS PROHIBITED.

GENERAL NOTES:

1. MATERIAL: 303 STAINLESS STEEL

2. (delta) BUSHING FIT WITH CROSS ARM AT 37°C 100% HUMIDITY

REV	MODEL BY	DRAWN BY	CHECKED BY	DATE
A	SAMIR SHREIM	SAMIR SHREIM	SAMIR SHREIM	5/21/2009
B	SAMIR SHREIM	SAMIR SHREIM		

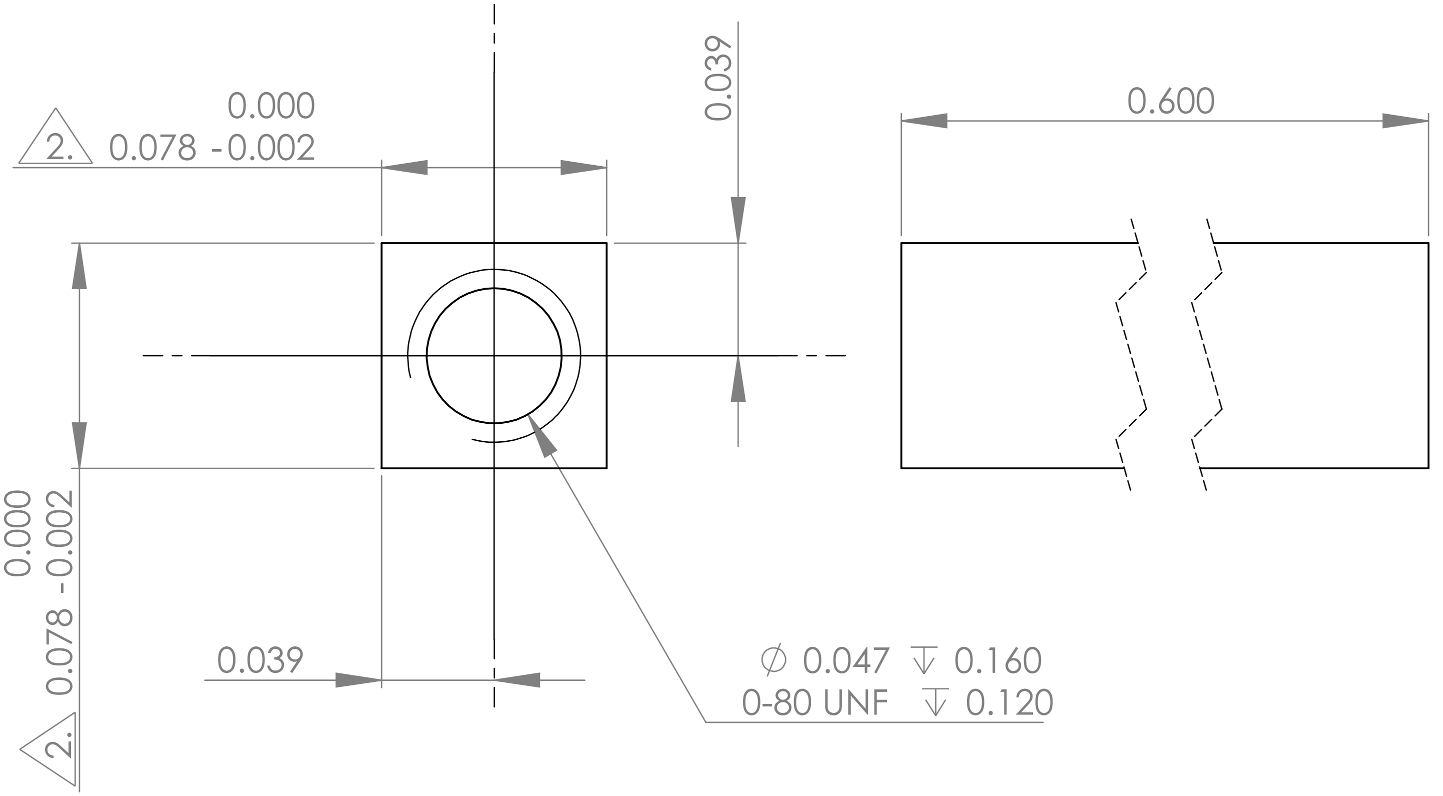

UNLESS OTHERWISE SPECIFIED
DIMENSIONS ARE IN INCHES
TOLERANCES:
FRACTIONAL ± 1/32
ANGULAR: MACH ± 0.5 DEG
TWO PLACE DECIMAL ±0.01
THREE PLACE DECIMAL ±0.005

TITLE:

LEVER ARM

PROPRIETARY AND CONFIDENTIAL
THE INFORMATION CONTAINED IN THIS DRAWING IS THE SOLE PROPERTY OF THE BEAMS LAB AT UCI'S BECKMAN LASER INSTITUTE. ANY REPRODUCTION IN PART OR AS A WHOLE WITHOUT THE WRITTEN PERMISSION OF THE BEAMS LAB IS PROHIBITED.

SIZE	DWG. NO.	REV
A	107-001-002	B

SCALE: 15:1 WEIGHT: SHEET 1 OF 1

5 4 3 2 1

GENERAL NOTES:

1. MATERIAL: IMPLANT GRADE UHMWPE

REV	MODEL BY	DRAWN BY	CHECKED BY	DATE
A	SAMIR SHREIM	SAMIR SHREIM	SAMIR SHREIM	5/21/2009
B	SAMIR SHREIM	SAMIR SHREIM		

2. (triangle) BUSHING FIT WITH CROSS ARM AT 37°C 100% HUMIDITY

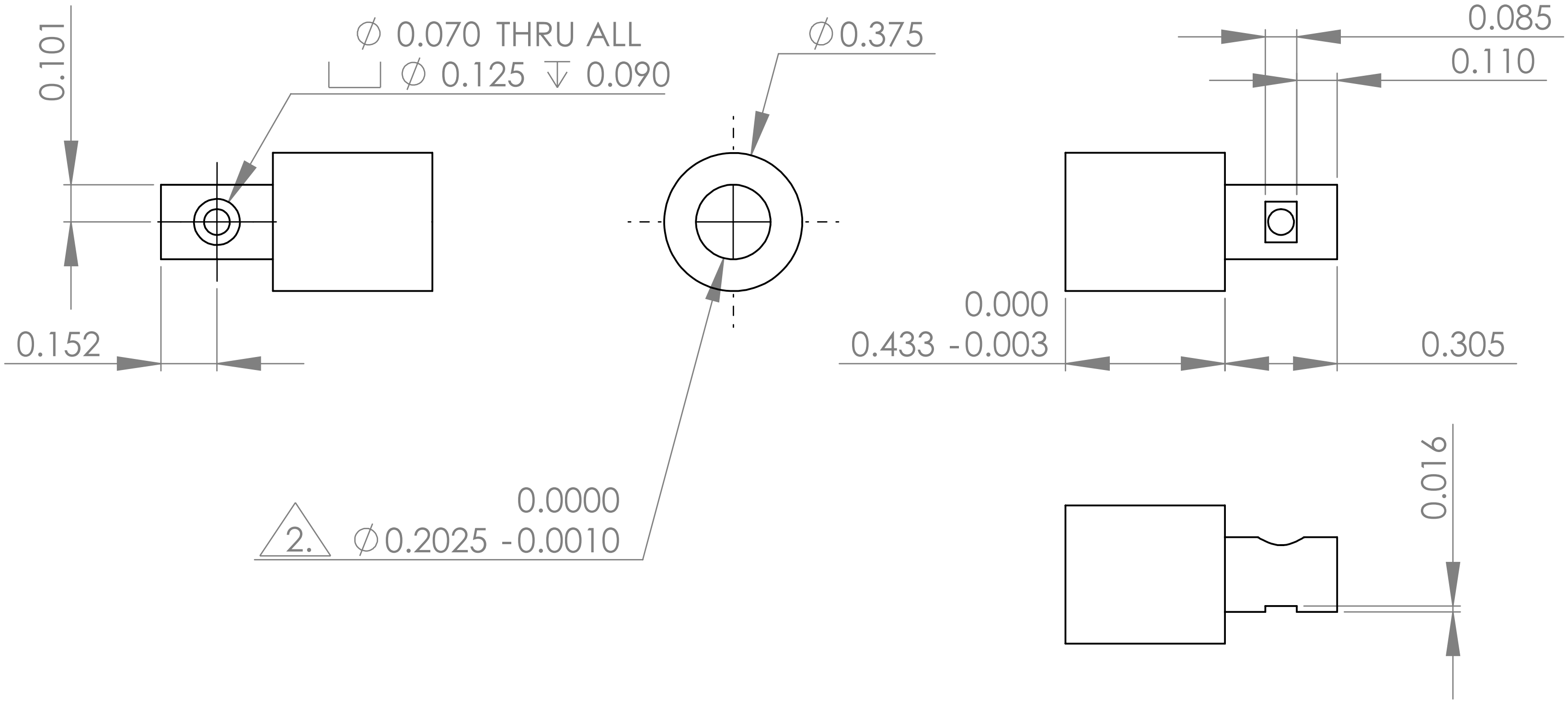

UNLESS OTHERWISE SPECIFIED<br>DIMENSIONS ARE IN INCHES<br>TOLERANCES:<br>FRACTIONAL ± 1/32<br>ANGULAR: MACH ± 0.5 DEG<br>TWO PLACE DECIMAL ±0.01<br>THREE PLACE DECIMAL ±0.005	TITLE:<br>ROTATOR SHAFT	
SIZE A	DWG. NO. 107-001-003	REV B
SCALE: 3:1	WEIGHT:	SHEET 1 OF 1

PROPRIETARY AND CONFIDENTIAL

THE INFORMATION CONTAINED IN THIS DRAWING IS THE SOLE PROPERTY OF THE BEAMS LAB AT UCI'S BECKMAN LASER INSTITUTE. ANY REPRODUCTION IN PART OR AS A WHOLE WITHOUT THE WRITTEN PERMISSION OF THE BEAMS LAB IS PROHIBITED.

5 4 3 2 1

GENERAL NOTES:

1. LUBRICATE THREADS WITH DOW CORNING 360 MEDICAL FLUID 1000 CST (POLYDIMETHYLSILOXANE)

REV	MODEL BY	DRAWN BY	CHECKED BY	DATE
A	SAMIR SHREIM	SAMIR SHREIM	SAMIR SHREIM	5/21/2009
B	SAMIR SHREIM	SAMIR SHREIM	SAMIR SHREIM	3/24/2010
C	SAMIR SHREIM	SAMIR SHREIM		

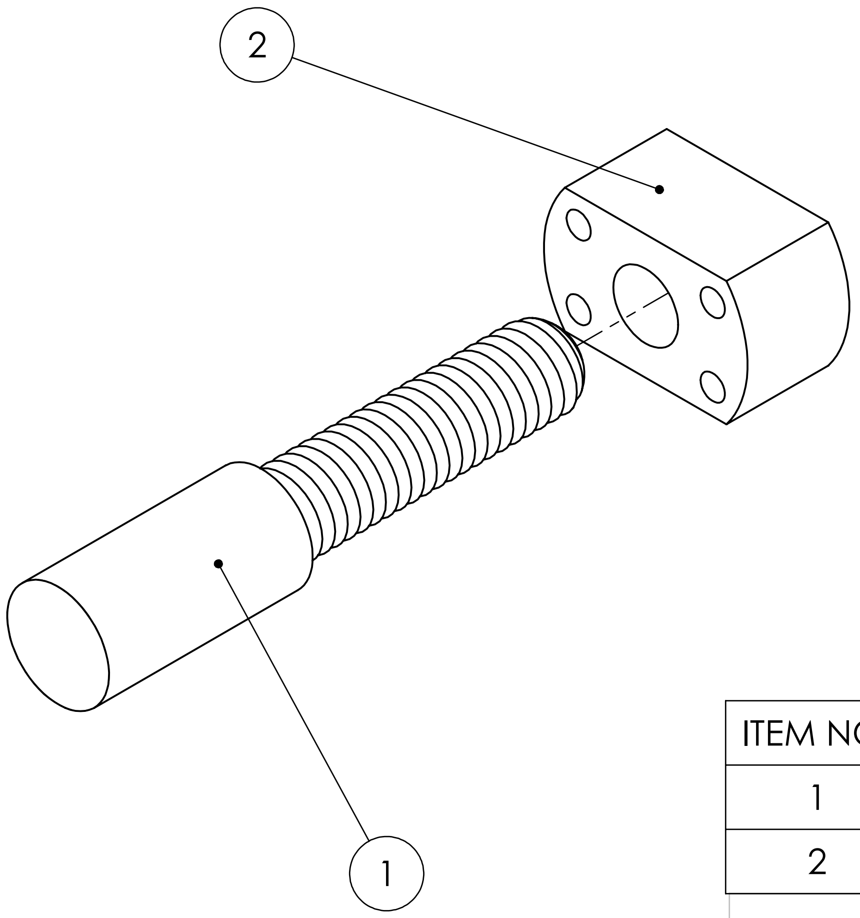

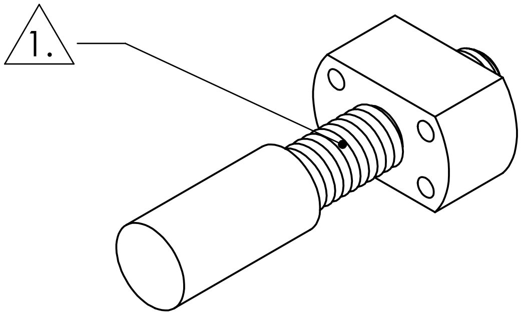

ITEM NO.	PART NUMBER	DESCRIPTION	QTY.
1	107-002-001	LEADSCREW	1
2	107-002-002	LEADSCREW BLOCK	1

UNLESS OTHERWISE SPECIFIED
DIMENSIONS ARE IN INCHES
TOLERANCES:
FRACTIONAL ± 1/32
ANGULAR: MACH ± 0.5 DEG
TWO PLACE DECIMAL ±0.01
THREE PLACE DECIMAL ±0.005

TITLE:

LEADSCREW ASSEMBLY

PROPRIETARY AND CONFIDENTIAL
THE INFORMATION CONTAINED IN THIS DRAWING IS THE SOLE PROPERTY OF THE BEAMS LAB AT UCI'S BECKMAN LASER INSTITUTE. ANY REPRODUCTION IN PART OR AS A WHOLE WITHOUT THE WRITTEN PERMISSION OF THE BEAMS LAB IS PROHIBITED.

SIZE	DWG. NO.	REV
A	107-002-000	C

SCALE: 3:1 | WEIGHT: | SHEET 1 OF 1

5 4 3 2 1

GENERAL NOTES:

1. MATERIAL: MORTON MACHINE WORKS P/N 4360 (303 STAINLESS STEEL)

REV	MODEL BY	DRAWN BY	CHECKED BY	DATE
A	SAMIR SHREIM	SAMIR SHREIM		

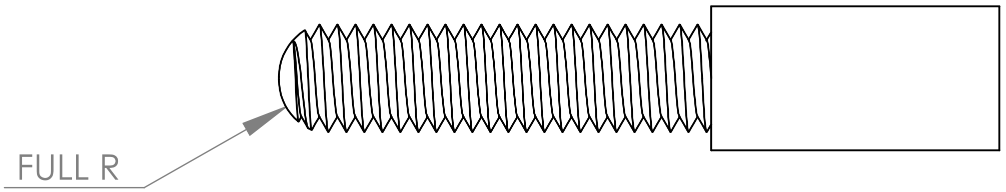

UNLESS OTHERWISE SPECIFIED
DIMENSIONS ARE IN INCHES
TOLERANCES:
FRACTIONAL ± 1/32
ANGULAR: MACH ± 0.5 DEG
TWO PLACE DECIMAL ±0.01
THREE PLACE DECIMAL ±0.005

TITLE:

LEADSCREW

PROPRIETARY AND CONFIDENTIAL
THE INFORMATION CONTAINED IN THIS DRAWING IS THE SOLE PROPERTY OF THE BEAMS LAB AT UCI'S BECKMAN LASER INSTITUTE. ANY REPRODUCTION IN PART OR AS A WHOLE WITHOUT THE WRITTEN PERMISSION OF THE BEAMS LAB IS PROHIBITED.

SIZE	DWG. NO.	REV
A	107-002-001	A

SCALE: 3:1 WEIGHT: SHEET 1 OF 1

5 4 3 2 1

GENERAL NOTES:

1. MATERIAL: 6061 ALUMINIUM

REV	MODEL BY	DRAWN BY	CHECKED BY	DATE
A	SAMIR SHREIM	SAMIR SHREIM	SAMIR SHREIM	5/21/2009
B	SAMIR SHREIM	SAMIR SHREIM	SAMIR SHREIM	3/24/2010
C	SAMIR SHREIM	SAMIR SHREIM		

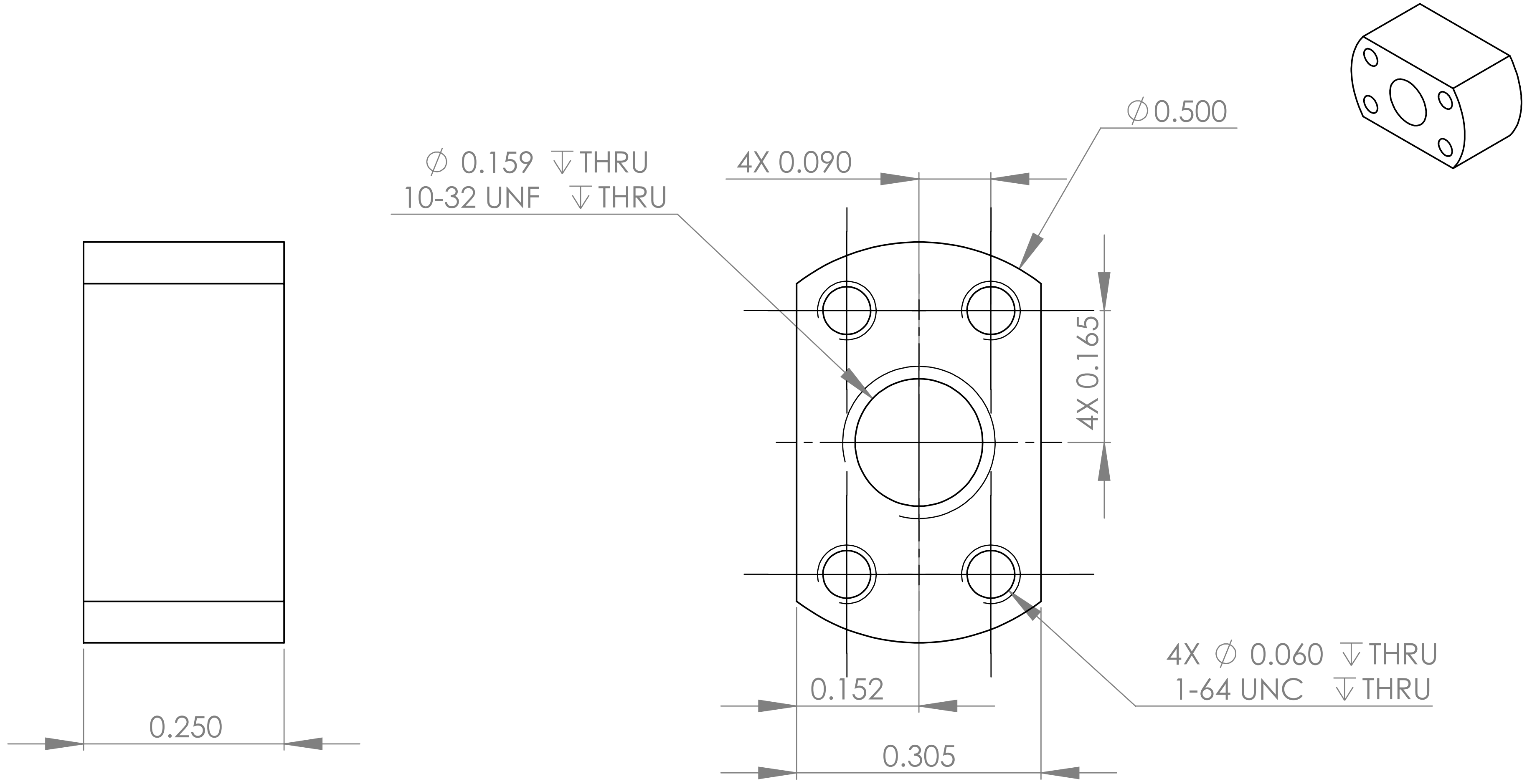

UNLESS OTHERWISE SPECIFIED
DIMENSIONS ARE IN INCHES
TOLERANCES:
FRACTIONAL ± 1/32
ANGULAR: MACH ± 0.5 DEG
TWO PLACE DECIMAL ±0.01
THREE PLACE DECIMAL ±0.005

TITLE:

LEADSCREW BLOCK

PROPRIETARY AND CONFIDENTIAL
THE INFORMATION CONTAINED IN THIS DRAWING IS THE SOLE PROPERTY OF THE BEAMS LAB AT UCI'S BECKMAN LASER INSTITUTE. ANY REPRODUCTION IN PART OR AS A WHOLE WITHOUT THE WRITTEN PERMISSION OF THE BEAMS LAB IS PROHIBITED.

SIZE A | DWG. NO. 107-002-002 | REV C

SCALE: 5:1 | WEIGHT: | SHEET 1 OF 1

5 4 3 2 1

GENERAL NOTES:

1. BUSHING FIT AT 37°C 100% HUMIDITY

REV	MODEL BY	DRAWN BY	CHECKED BY	DATE
A	SAMIR SHREIM	SAMIR SHREIM	SAMIR SHREIM	5/21/2009
B	SAMIR SHREIM	SAMIR SHREIM		

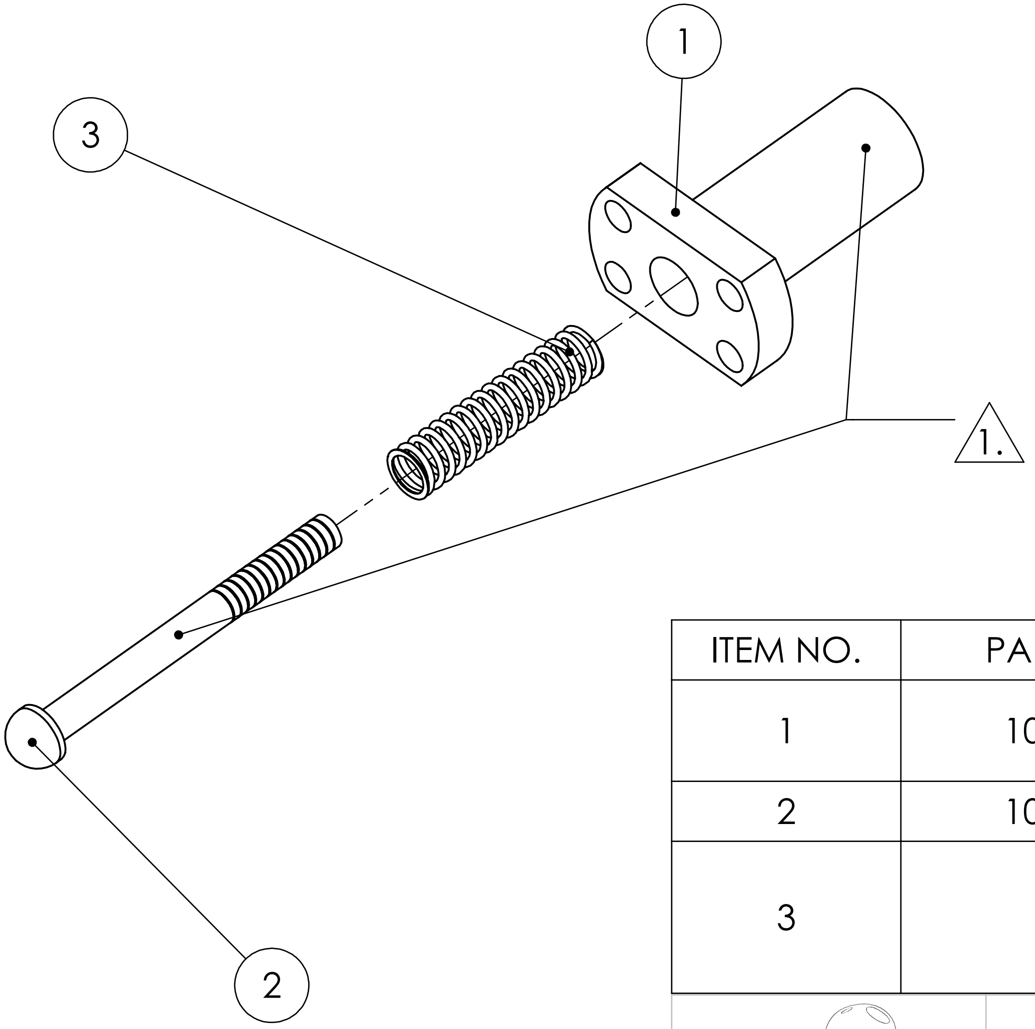

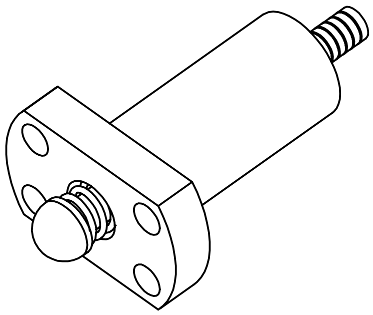

ITEM NO.	PART NUMBER	DESCRIPTION	QTY.
1	107-003-001	SPRING PLUNGER BLOCK	1
2	107-003-002	SPRING PLUNGER	1
3	-	SPRING, CENTURY SPRING CORP. P/N 70100S	1

UNLESS OTHERWISE SPECIFIED
DIMENSIONS ARE IN INCHES
TOLERANCES:
FRACTIONAL ± 1/32
ANGULAR: MACH ± 0.5 DEG
TWO PLACE DECIMAL ±0.01
THREE PLACE DECIMAL ±0.005

TITLE:

SPRING PLUNGER ASSEMBLY

PROPRIETARY AND CONFIDENTIAL
THE INFORMATION CONTAINED IN THIS DRAWING IS THE SOLE PROPERTY OF THE BEAMS LAB AT UCI'S BECKMAN LASER INSTITUTE. ANY REPRODUCTION IN PART OR AS A WHOLE WITHOUT THE WRITTEN PERMISSION OF THE BEAMS LAB IS PROHIBITED.

SIZE A | DWG. NO. 107-003-000 | REV B

SCALE: 2.5:1 WEIGHT: | SHEET 1 OF 1

5 4 3 2 1

GENERAL NOTES:

1. MATERIAL: ACETAL COPOLYMER BLACK

REV	MODEL BY	DRAWN BY	CHECKED BY	DATE
A	SAMIR SHREIM	SAMIR SHREIM	SAMIR SHREIM	5/21/2009
B	SAMIR SHREIM	SAMIR SHREIM		

2. BUSHING FIT WITH SPRING PLUNGER AT 37°C 100% HUMIDITY

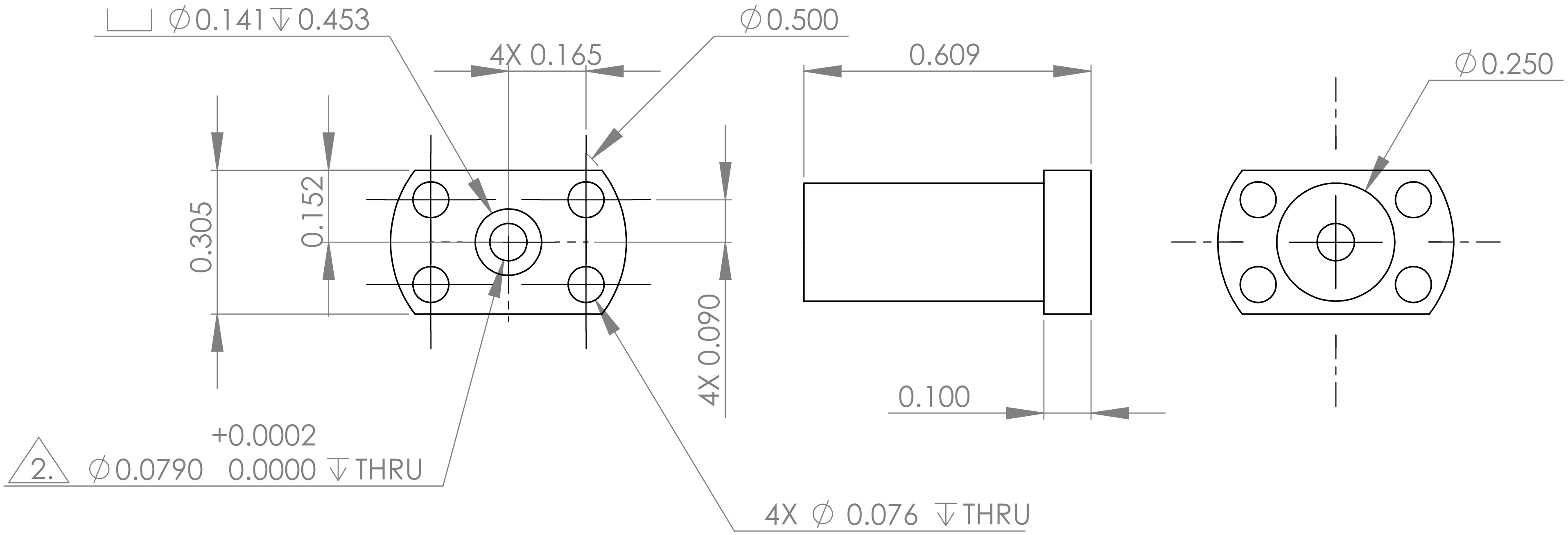

UNLESS OTHERWISE SPECIFIED
DIMENSIONS ARE IN INCHES
TOLERANCES:
FRACTIONAL ± 1/32
ANGULAR: MACH ± 0.5 DEG
TWO PLACE DECIMAL ±0.01
THREE PLACE DECIMAL ±0.005

TITLE:

SPRING PLUNGER BLOCK

PROPRIETARY AND CONFIDENTIAL
THE INFORMATION CONTAINED IN THIS DRAWING IS THE SOLE PROPERTY OF THE BEAMS LAB AT UCI'S BECKMAN LASER INSTITUTE. ANY REPRODUCTION IN PART OR AS A WHOLE WITHOUT THE WRITTEN PERMISSION OF THE BEAMS LAB IS PROHIBITED.

SIZE	DWG. NO.	REV
A	107-003-001	B

SCALE: 3:1 WEIGHT: SHEET 1 OF 1

5 4 3 2 1

GENERAL NOTES:

1. MATERIAL: 303 STAINLESS STEEL

REV	MODEL BY	DRAWN BY	CHECKED BY	DATE
A	SAMIR SHREIM	SAMIR SHREIM	SAMIR SHREIM	5/21/2009
B	SAMIR SHREIM	SAMIR SHREIM	SAMIR SHREIM	

△2. BUSHING FIT WITH SPRIN PLUNGER BLOCK AT 37°C 100% HUMIDITY

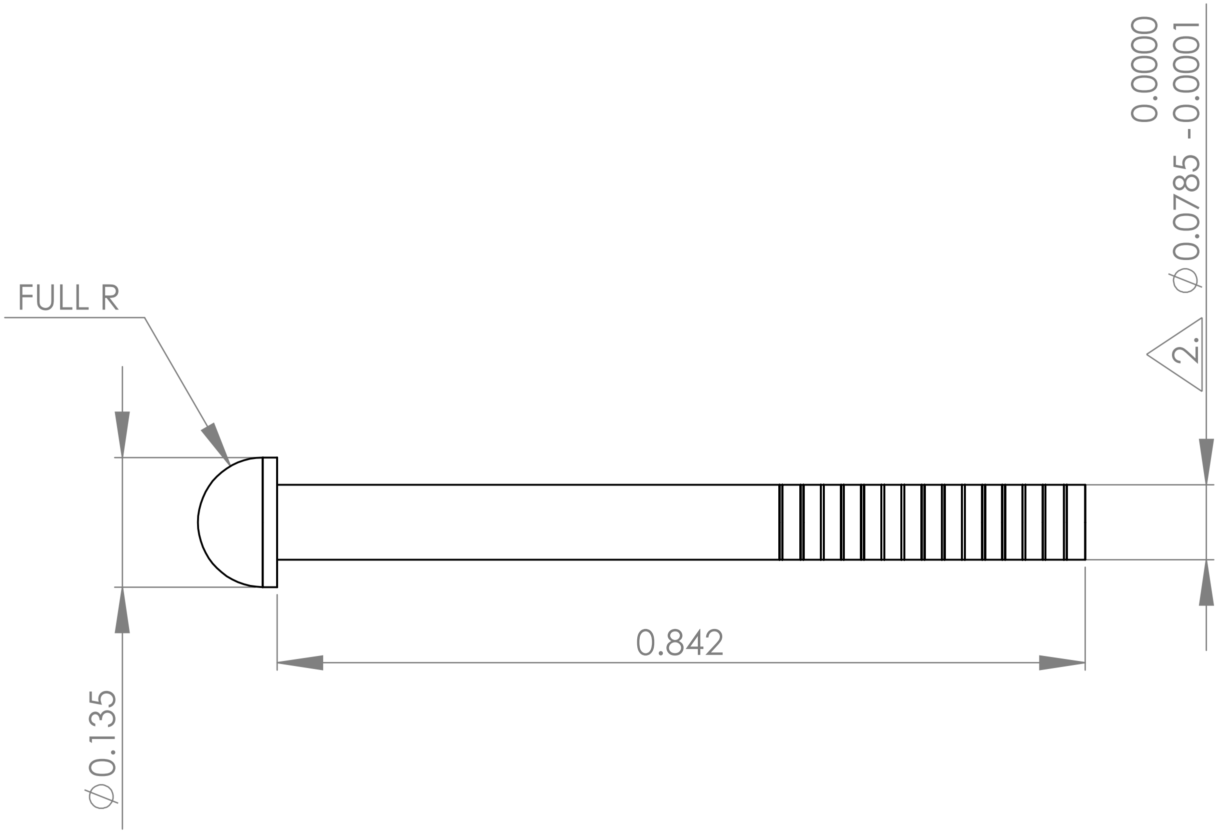

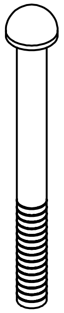

UNLESS OTHERWISE SPECIFIED	TITLE:
DIMENSIONS ARE IN INCHES<br>TOLERANCES:<br>FRACTIONAL ±1/32<br>ANGULAR: MACH ± 0.5 DEG<br>TWO PLACE DECIMAL ±0.01<br>THREE PLACE DECIMAL ±0.005	SPRING PLUNGER

PROPRIETARY AND CONFIDENTIAL
THE INFORMATION CONTAINED IN THIS DRAWING IS THE SOLE PROPERTY OF THE BEAMS LAB AT UCI'S BECKMAN LASER INSTITUTE. ANY REPRODUCTION IN PART OR AS A WHOLE WITHOUT THE WRITTEN PERMISSION OF THE BEAMS LAB IS PROHIBITED.

SIZE	DWG. NO.	REV
A	107-003-002	B
SCALE: 5:1	WEIGHT:	SHEET 1 OF 3

5 4 3 2 1

GENERAL NOTES:

1. CENTER MARK

2. LASER MARKED PER DIAGRAM ON SHEET 3 (VENDOR: LASERMATION INC.)

3. LASER MARK PRIOR TO TURNING RADIUS

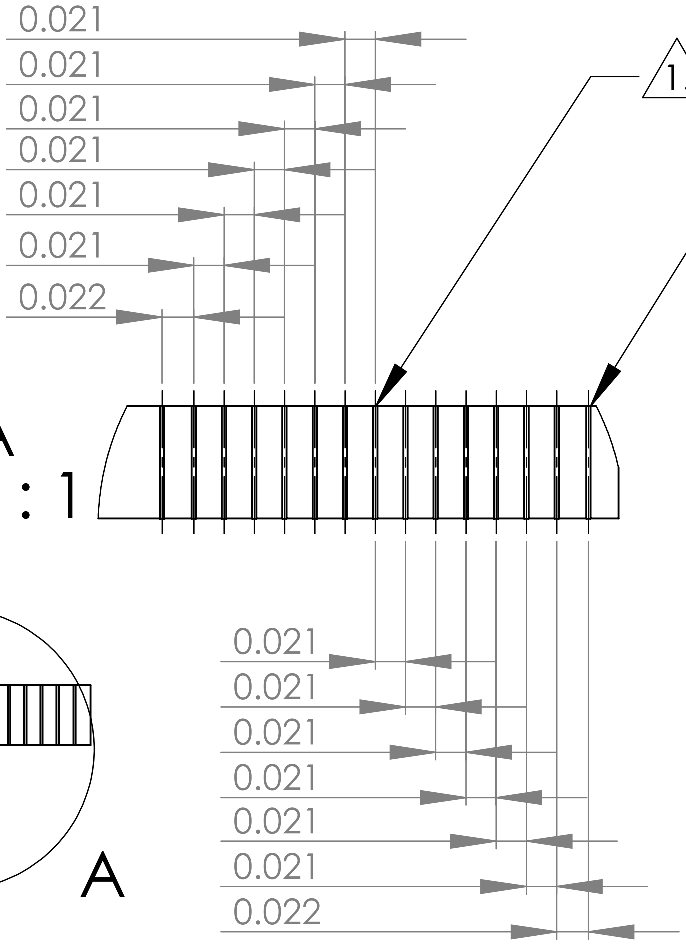

DETAIL A
SCALE 7.5 : 1

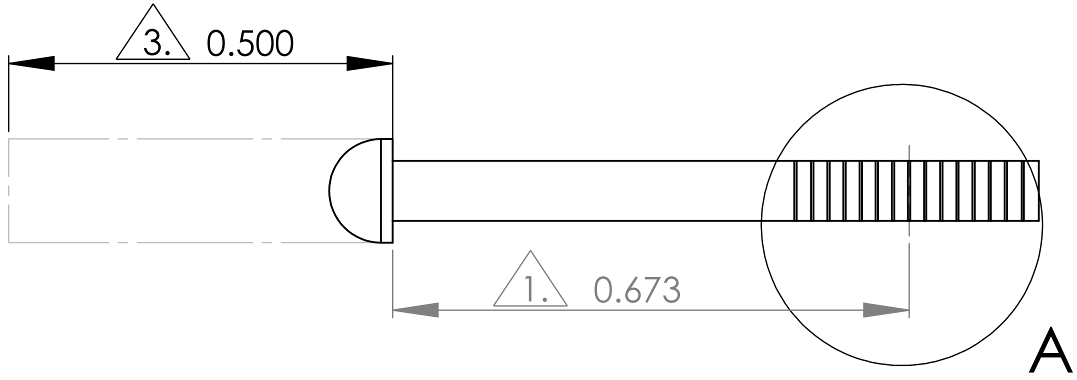

UNLESS OTHERWISE SPECIFIED	TITLE:
DIMENSIONS ARE IN INCHES<br>TOLERANCES:<br>FRACTIONAL ± 1/32<br>ANGULAR: MACH ± 0.5 DEG<br>TWO PLACE DECIMAL ±0.01<br>THREE PLACE DECIMAL ±0.005	SPRING PLUNGER

PROPRIETARY AND CONFIDENTIAL
THE INFORMATION CONTAINED IN THIS DRAWING IS THE SOLE PROPERTY OF THE BEAMS LAB AT UCI'S BECKMAN LASER INSTITUTE. ANY REPRODUCTION IN PART OR AS A WHOLE WITHOUT THE WRITTEN PERMISSION OF THE BEAMS LAB IS PROHIBITED.

SIZE	DWG. NO.	REV
A	107-003-002	B
SCALE: 4:1	WEIGHT:	SHEET 2 OF 3

5 4 3 2 1

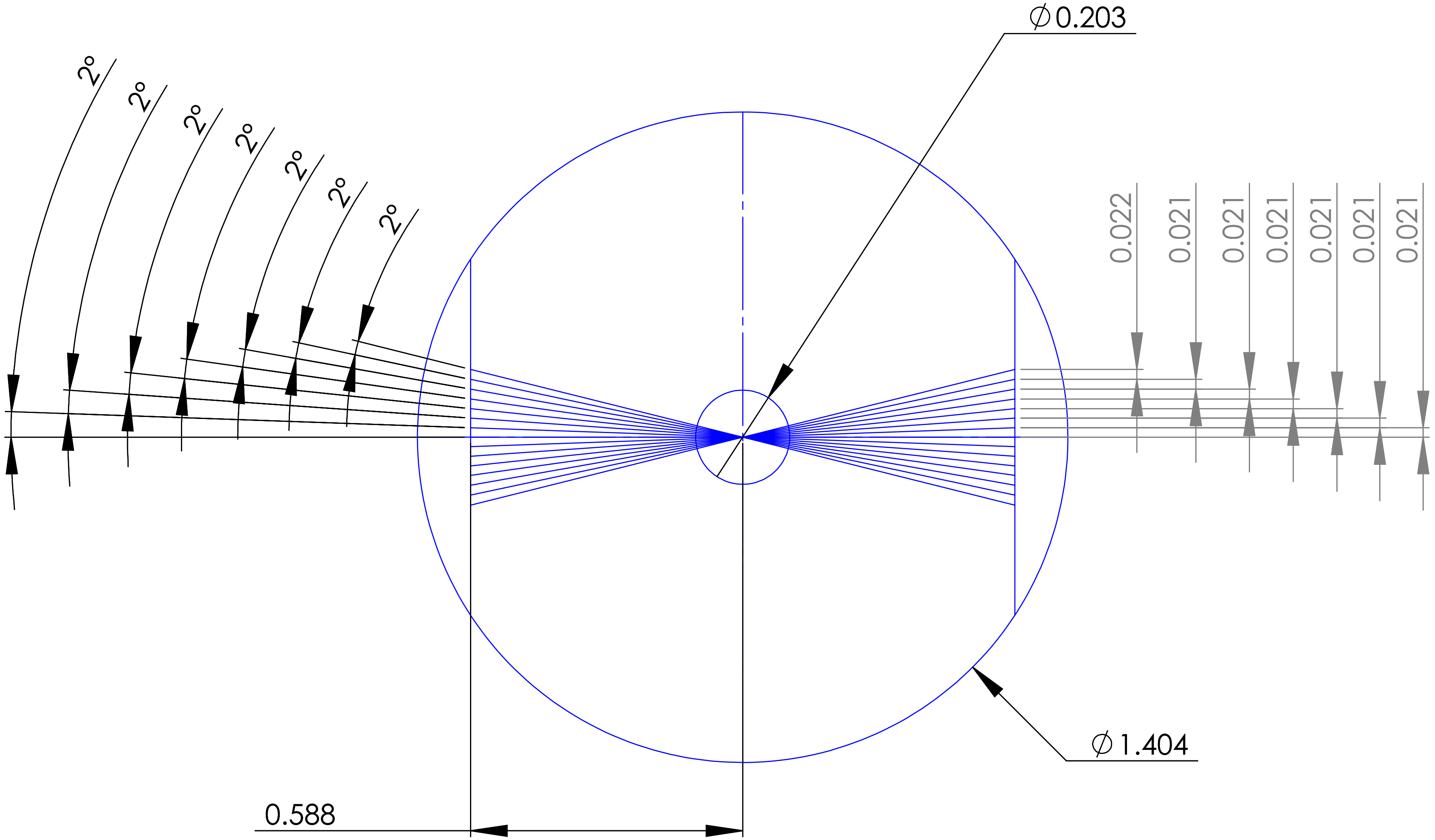

UNLESS OTHERWISE SPECIFIED
DIMENSIONS ARE IN INCHES
TOLERANCES:
FRACTIONAL ± 1/32
ANGULAR: MACH ± 0.5 DEG
TWO PLACE DECIMAL ±0.01
THREE PLACE DECIMAL ±0.005

TITLE:

SPRING PLUNGER

PROPRIETARY AND CONFIDENTIAL
THE INFORMATION CONTAINED IN THIS DRAWING IS THE SOLE PROPERTY OF THE BEAMS LAB AT UCI'S BECKMAN LASER INSTITUTE. ANY REPRODUCTION IN PART OR AS A WHOLE WITHOUT THE WRITTEN PERMISSION OF THE BEAMS LAB IS PROHIBITED.

SIZE	DWG. NO.	REV
A	107-003-002	B

SCALE: 3:1 | WEIGHT: | SHEET 3 OF 3

5 4 3 2 1
